# MARCH5 regulates mitotic apoptosis through MCL1-dependent and independent mechanisms

**DOI:** 10.1038/s41418-022-01080-2

**Published:** 2022-11-03

**Authors:** Yang Wang, Randy Y. C. Poon

**Affiliations:** 1 Division of Life Science, Hong Kong University of Science and Technology, Clear Water Bay, Hong Kong, China; 2 State Key Laboratory of Molecular Neuroscience, Hong Kong University of Science and Technology, Clear Water Bay, Hong Kong, China

**Keywords:** Proteasome, Ubiquitin ligases

## Abstract

The anti-apoptotic MCL1 is critical for delaying apoptosis during mitotic arrest. MCL1 is degraded progressively during mitotic arrest, removing its anti-apoptotic function. We found that knockout of components of ubiquitin ligases including APC/C, SCF complexes, and the mitochondrial ubiquitin ligase MARCH5 did not prevent mitotic degradation of MCL1. Nevertheless, MARCH5 determined the initial level of MCL1–NOXA network upon mitotic entry and hence the window of time during MCL1 was present during mitotic arrest. Paradoxically, although knockout of MARCH5 elevated mitotic MCL1, mitotic apoptosis was in fact enhanced in a BAK-dependent manner. Mitotic apoptosis was accelerated after MARCH5 was ablated in both the presence and absence of MCL1. Cell death was not altered after disrupting other MARCH5-regulated BCL2 family members including NOXA, BIM, and BID. Disruption of the mitochondrial fission factor DRP1, however, reduced mitotic apoptosis in MARCH5-disrupted cells. These data suggest that MARCH5 regulates mitotic apoptosis through MCL1-independent mechanisms including mitochondrial maintenance that can overcome the stabilization of MCL1.

## Introduction

Antimitotic drugs are cornerstones in conventional cancer therapies. These diverse classes of drugs share a common function in trapping cells in protracted mitosis. The consequence cell death occurring either during the mitotic arrest or after aberrant mitotic exit such as mitotic slippage is generally termed mitotic catastrophe [1]. The molecular basis of mitotic catastrophe can be understood in part in the context of normal mitosis. As cyclin B–CDK1 complexes are essential components of the mitotic engine, destruction of cyclin B by the anaphase-promoting complex/cyclosome (APC/C) is a key event triggering mitotic exit [2]. During early mitosis, APC/C is inhibited by the spindle-assembly checkpoint (SAC), which senses unattached or improperly attached kinetochores [3]. As APC/C is active only after SAC is satisfied, antimitotic drugs that disrupt spindle dynamics can stall mitosis in a prometaphase-like state [4]. Nevertheless, the fate of individual cells after protracted mitotic arrest varies greatly [5]. On the one hand, the accumulation of apoptotic activators and/or a loss of apoptotic inhibitors can induce mitotic apoptosis. On the other hand, mitotic exit can occur without proper chromosome segregation in a process termed mitotic slippage. Whether a cell dies or undergoes mitotic slippage is the consequence of which of the thresholds of apoptosis or mitotic slippage is breached first.

The current paradigm states that mitotic slippage is caused by a gradual degradation of cyclin B, which is in part due to the weakening of the SAC during prolonged mitotic block [6]. On the other hand, the underlying mechanisms that promote apoptotic signals during mitosis remains incompletely understood. Members of the BCL-2 family are particularly interesting in this process because many of them are known to be modified during mitosis. Functionally, members of BCL-2 family can be divided into initiators, effectors, and anti-apoptotic proteins [7]. The initiators (including BAD, BID, BIK, BIM, BMF, HRK, PUMA and NOXA) are BH3-only proteins that transduce pro-apoptotic signals by either neutralizing anti-apoptotic BCL-2 proteins or by directly activating pro-apoptotic effectors. Both effectors (BAX, BAK and probably BOK) and anti-apoptotic proteins are multi-BH domain proteins with similar globular structures. The pro-apoptotic function of the effectors is mediated through the induction of mitochondrial outer membrane permeabilization (MOMP), which is antagonized by anti-apoptotic BCL-2 proteins (at least six members are present in human: BCL-2, BCL-XL, BCL-W, A1, BCL-B, and MCL1).

Unlike other members of the BCL-2 family, MCL1 (Myeloid cell leukemia-1) is unstable during mitotic arrest [8]. MCL1 degradation is dependent on another BCL2-like protein NOXA [9]. The progressive destruction of MCL1 can potentially shift the balance of apoptotic signals during mitotic block to favor apoptosis. Factors that alter the initial levels or rate of mitotic degradation MCL1 can potentially affect the sensitivity of antimitotic drugs.

Which ubiquitin ligase(s) is responsible for MCL1 degradation during mitosis is controversial. Several ubiquitin ligases have been established to target MCL1 during interphase, including βTrCP [10], FBW7 [11, 12], TRIM17 [13], and HUWE1 (MULE) [14, 15]. Furthermore, APC/C has also been implicated to target MCL1 for degradation in a cyclin B1–CDK1-dependent manner [16].

A mitochondrial ubiquitin ligase called MARCH5 (also called MARCHF5 and MITOL) has recently been implicated in controlling the stability of MCL1. MARCH5 is located at the outer mitochondrial membrane with a cytoplasmatic RING finger domain. Its best documented role is in the regulation of mitochondrial fission and fusion in response to mitochondrial stress [17–22]. MARCH5 has been demonstrated to target MCL1 for degradation upon several types of stresses in a NOXA-dependent manner [23–26]. Importantly, downregulation of MARCH5 not only increases MCL1 during mitosis, but sensitizes cells to apoptosis caused by antimitotic drugs, both during the mitotic arrest and upon mitotic slippage following prolonged block in mitosis [26].

In this study, we addressed the contribution of MCL1 in MARCH5-controlled cell death during mitosis. We found that although knockout of MARCH5 stabilized MCL1 during interphase and to some extent during mitosis, it did not prevent the degradation of MCL1 during mitotic arrest. Despite the overall increase of mitotic MCL1, mitotic apoptosis was in fact accelerated in MARCH5-deficient cells in a BAK-dependent manner. These results revealed that targets in addition to MCL1, including proteins involving in mitochondrial maintenance, are responsible for MARCH5-controlled mitotic apoptosis.

## Results

### Differential roles of APC/C, SCF, and MARCH5 ubiquitin ligases in setting thresholds of MCL1 during mitosis

**Fig. 1:**
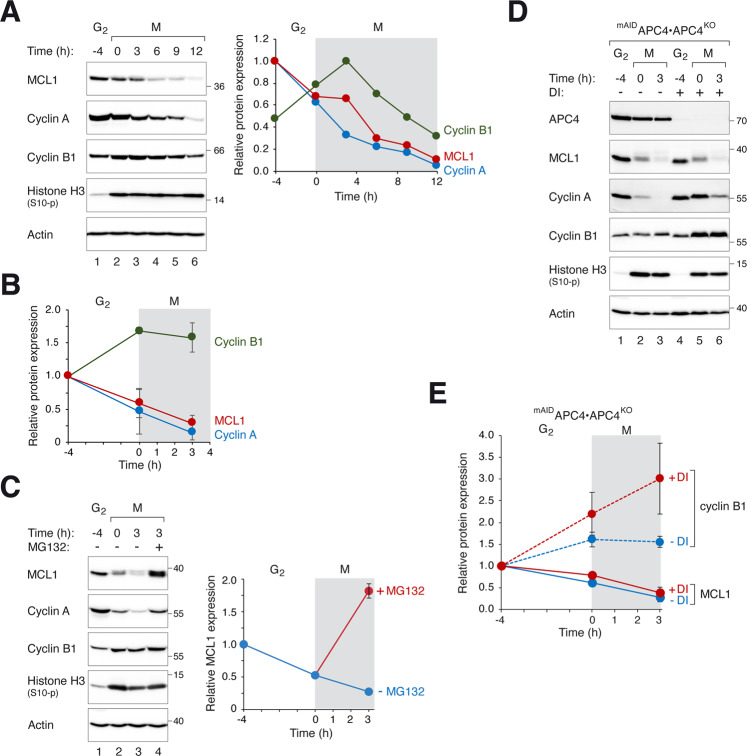
Proteasome-dependent degradation of MCL1 during mitotic arrest. **A** Stability of MCL1 during mitotic arrest. HeLa cells were synchronized using a double thymidine procedure. G_2_ samples were harvested at 8 h after release from the second thymidine block (indicated as *t* = −4 h). Cells were trapped in mitosis (M) using NOC and isolated by shake off (*t* = 0) before further incubated with NOC. Cells were harvested at different time points. Lysates were prepared and analyzed with immunoblotting. Actin analysis was included to assess protein loading and transfer. Phosphorylated histone H3^Ser10^ is a marker of mitosis. The intensity of the bands of MCL1, cyclin A, and cyclin B1 was quantified (right-hand panel). **B** Mitotic arrest stabilizes cyclin B1 and destabilizes MCL1. Cells were synchronized as described in **A**. The expression of MCL1, cyclin A, and cyclin B1 during G_2_ and mitosis was quantified from three independent experiments (mean ± SEM). **C** Mitotic degradation of MCL1 is proteasome-dependent. Cells were synchronized and trapped in mitosis as described above. Mitotic cells were exposed to either buffer or MG132 and harvested after 3 h. The expression of MCL1 was analyzed with immunoblotting. The MCL1 band intensity was quantified (mean ± SEM from three independent experiments). **D** MCL1 is degraded during mitosis by an APC/C-independent mechanism. APC4^KO^ cells expressing ^mAID^APC4 were generated. The cells were synchronized and arrested in mitosis as before. DI were applied to turn off the expression of ^mAID^APC4 at the time of second thymidine release. Lysates were prepared and analyzed with immunoblotting. **E** Disruption of APC4 stabilizes cyclin B1 but not MCL1. Synchronization experiments were performed using ^mAID^APC4-expressing APC4^KO^ cells as described in **D**. The MCL1 and cyclin B1 bands were quantified and shown in the right-hand panels (normalized to G_2_ expression). Mean ± SEM of three independent experiments.

Mitotic cells were obtained by synchronizing cells with a double thymidine procedure before exposing G_2_ cells with the microtubule-disrupting agent nocodazole (NOC). Mitotic cells were then isolated and incubated further with NOC-containing medium (Fig. 1A). Mitotic arrest was confirmed by the accumulation of cyclin B1 and histone H3^Ser10^ phosphorylation. The expression of MCL1 was already reduced during early mitosis compared to G_2_. Similar to the behavior of cyclin A [27], MCL1 expression was further reduced during SAC-dependent mitotic arrest (Fig. 1B). Furthermore, the turnover of MCL1 was proteasome-dependent, as it could be prevented with MG132 (Fig. 1C).

We next examined potential ubiquitin ligases that regulate MCL1 degradation during mitotic entry and arrest. As the mitotic ubiquitin ligase APC/C^CDC20^ has been implicated for MCL1 degradation [16], we first examined MCL1 stability by using a CDC20^KO^ cell line (CDC20 was ablated using CRISPR-Cas9) expressing ^HA^CDC20 under the control of an inducible promoter. Supplementary Fig. S1A shows that unlike cyclin A and cyclin B1, which were stabilized after ^HA^CDC20 was turned off, MCL1 was degraded during mitosis. We were also able to detect cyclin A–CDC20, but not MCL1–CDC20 interaction, using immunoprecipitation (Fig. S1B). Finally, we also generated a conditional APC4 (a core subunit of APC/C)-depleted cell line. APC4 fused with a mini auxin-inducible degron (mAID) was expressed in cells of which the endogenous APC4 gene was ablated using CRISPR-Cas9. As ^mAID^APC4 was placed under the control of a Tet-Off promoter, it could be turned off rapidly with doxycycline (to turn off transcription) and indole-3-acetic acid (to degrade the degron) together (DI herein) [28]. As expected, cyclin A and cyclin B1 were stabilized in the absence of APC4. By contrast, MCL1 was degraded in the absence of APC4 (Fig. 1D, E). Taken together, these data indicated that MCL1 degradation during mitotic arrest is not carried out by APC/C^CDC20^.

SCF complexes have also been implicated as ubiquitin ligases for MCL1 [10–12]. We examined the involvement of SCF complexes in mitotic MCL1 stability by depleting the SCF core component SKP1. Using SKP1^KO^ cells expressing ^FLAG^SKP1, we found that mitotic MCL1 was unstable after ^FLAG^SKP1 was turned off, suggesting that mitotic MCL1 degradation was SCF-independent (Fig. S1C). As a control, cyclin E was stabilized in the absence of SKP1 [29].

**Fig. 2:**
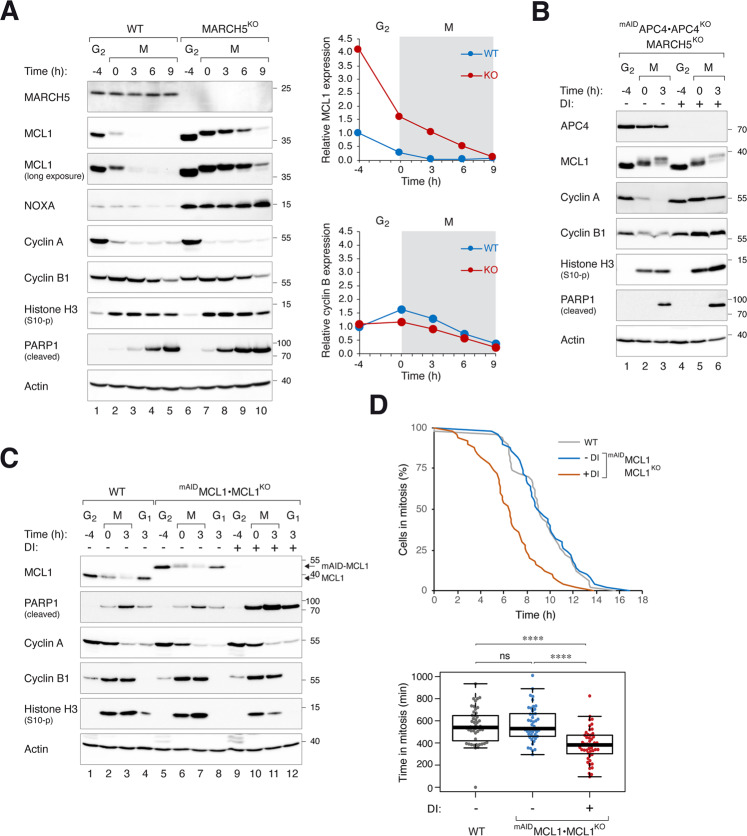
MARCH5 determines MCL1 expression at mitotic entry. **A** KO of MARCH5 elevates MCL1 during mitotic arrest. Parental HeLa (WT) and MARCH5^KO^ cells were synchronized and arrested in mitosis as before. Protein expression was analyzed with immunoblotting. Different exposures of the MCL1 blot are shown to provide a better comparison of the degradation kinetics. The MCL1 and cyclin B1 bands were quantified and shown in the right-hand panels (normalized to G_2_ expression in HeLa cells). **B** Mitotic degradation of MCL1 is independent on MARCH5 and APC/C. APC4^KO^ expressing ^mAID^APC4 were generated in a MARCH5^KO^ background. The cells were synchronized and arrested in mitosis as before. DI were applied to turn off the expression of ^mAID^APC4 at the time of second thymidine release. Lysates were prepared and analyzed with immunoblotting. **C** Mitotic apoptosis is negatively regulated by MCL1. HeLa and MCL1^KO^ expressing ^mAID^MCL1 were synchronized and arrested in mitosis as before. ^mAID^MCL1 was turned off with DI at the time of second thymidine release. Protein expression was analyzed with immunoblotting. **D** Accelerated mitotic apoptosis in the absence of MCL1. HeLa and MCL1^KO^ expressing ^mAID^MCL1 were transiently transfected with histone H2B-GFP before synchronized and arrested in mitosis as before. The cells were either untreated or incubated with DI at the time of second thymidine release. Individual cells were tracked using live-cell imaging for 24 h (starting at 8 h after second thymidine release) (*n* = 50). The duration of mitotic arrest is plotted using Kaplan–Meier estimator. Box-and-whisker plots show the elapsed time between mitotic entry and mitotic apoptosis/slippage. ****p < 0.0001. The raw data for individual cells are shown in Fig. S6.

Given the evidence linking MCL1 stability with the mitochondrial ubiquitin ligase MARCH5 (see Introduction), we next examined MCL1 expression from G_2_ into mitosis in MARCH5-deficient cells. MARCH5 was undetectable after CRISPR-Cas9-mediated knockout (KO) (Fig. 2A). Gene-disrupting indels in the *MARCHF5* locus were verified by genome sequencing (Fig. S2). Ablation of MARCH5 resulted in an accumulation of MCL1 in interphase (Fig. 2A). Mitotic entry was unaffected, as indicated by the accumulation of phosphorylated histone H3^Ser10^. Significantly, MCL1 degradation still occurred upon mitotic entry and during mitotic arrest. Moreover, MCL1 was unstable in cells lacking both MARCH5 and APC4, indicating that the mitotic degradation of MCL1 still occurred in the absence of MARCH5 and APC/C (Fig. 2B). Nevertheless, the half-life of MCL1 during mitosis was longer in MARCH5^KO^ cells (Fig. S3). In combination with the elevated expression in G_2_, MCL1 expression was higher in MARCH5^KO^ than in WT during at least the initial 6 h of mitotic block. For a comparison, the cyclin B1 was unaffected by MARCH5.

Collectively, these data indicate that MARCH5 controls MCL1 stability during interphase and partially during mitotic arrest. Nevertheless, as MARCH5 determines the initial level of MCL1 upon mitotic entry, it has a major impact on the window of time during mitotic block of which MCL1 is present.

### MCL1 is critical for delaying mitotic apoptosis

To determine if MARCH5 could regulate mitotic apoptosis through MCL1, we first examined the contribution of MCL1 to the timing of apoptosis. As ablation of MCL1 compromised long-term survival in HeLa cells, we initially used a strategy in which MCL1 was ablated in a ^FLAG^BCL-XL-overexpressing background to prevent apoptosis (Fig. S4A). The ^FLAG^BCL-XL (under the control of an inducible promoter) was then turned off before the experiments. An increase in apoptosis was observed in MCL1-deficient cells compared to MCL1-containing cells, as indicated by the cleaved PARP1 signals (Fig. S4B). To ensure that the PARP1 cleavage in the assays was due to mitotic arrest and not interphase effects of NOC, a CDK1 inhibitor (RO3306) was added either before or after NOC to trap cells in G_2_ or induced mitotic slippage, respectively (Fig. S5). These analyses indicated that cleaved PARP1 only increased when cells were arrested in mitosis. Single cell analysis using live-cell imaging further verified that mitotic apoptosis was accelerated by several hours in the absence of MCL1 (Fig. S4C).

The above findings were further validated using a MCL1^KO^ cell line expressing ^mAID^MCL1. Degradation of ^mAID^MCL1 promoted mitotic apoptosis as indicated by PARP1 cleavage analysis (Fig. 2C) and live-cell imaging (Figs. 2D and S6). Consistent with the loss of anti-apoptotic activity due to proteasome-mediated degradation of MCL1, inhibition of MCL1 degradation using MG132 correlated with a reduction of apoptosis (Fig. S7). By contrast, MG132 did not prevent apoptosis in the absence of MCL1 (after ^mAID^MCL1 was turned off). Taken together, these data verified that in our experimental setting, the expression of MCL1 can determine the duration of mitotic arrest before the onset of apoptosis.

### MARCH5 is a critical determinant of mitotic apoptosis through both MCL1-dependent and -independent pathways

**Fig. 3:**
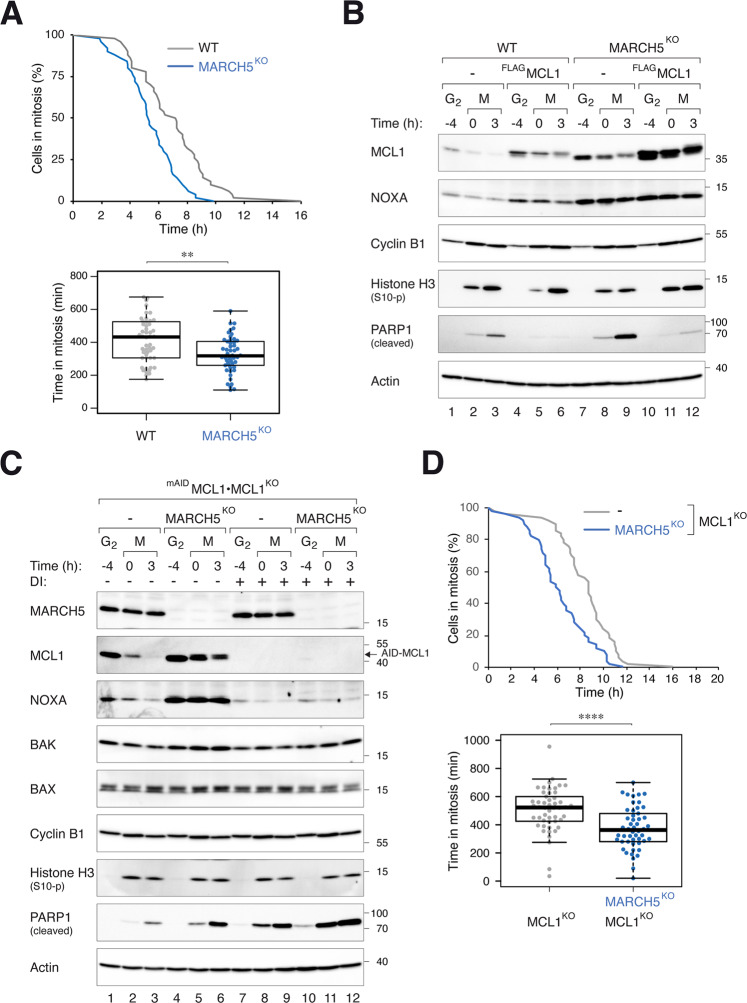
MARCH5 inhibits mitotic apoptosis through MCL1-dependent and -independent mechanisms. **A** Depletion of MARCH5 accelerates mitotic apoptosis. WT and MARCH5^KO^ cells were transfected with histone H2B-GFP before synchronized and arrested in mitosis as before. Individual cells were tracked using live-cell imaging for 24 h (starting at 6 h after second thymidine release) (*n* = 50). The duration of mitotic arrest is plotted using Kaplan-Meier estimator. Box-and-whisker plots show the elapsed time between mitotic entry and mitotic apoptosis/slippage. **p < 0.01. **B** Ectopic expression of MCL1 can abolish MARCH5^KO^-mediated mitotic apoptosis. Cells expressing ^FLAG^MCL1 were generated in HeLa or MARCH5^KO^ backgrounds. The cells were synchronized and arrested in mitosis as before. Protein expression was analyzed with immunoblotting. **C** Disruption of MCL1 promotes more apoptosis in MARCH5^KO^ cells. MARCH5 was inactivated with CRISPR-Cas9 in MCL1^KO^ expressing ^mAID^MCL1. The cells were synchronized and arrested in mitosis as before. ^mAID^MCL1 was turned off with DI. Protein expression was analyzed with immunoblotting. **D** Additive acceleration of mitotic apoptosis in the absence of MCL1 and MARCH5. MCL1^KO^ cells expressing ^mAID^MCL1 in either WT or MARCH5^KO^ background were transiently transfected with histone H2B-GFP before synchronized and arrested in mitosis as before. The cells were incubated with DI (to turn off MCL1) before individual cells were tracked using live-cell imaging for 24 h. The duration of mitotic arrest is plotted using Kaplan–Meier estimator. Box-and-whisker plots show the elapsed time between mitotic entry and mitotic apoptosis/slippage. ****p < 0.0001.

Consistent with findings from Haschka et al. [26], we found that mitotic apoptosis was exacerbated in the absence of MARCH5. This was verified by both an increase in PARP1 cleavage (Fig. 2A) and accelerated cell death (Fig. 3A). A similar increase in mitotic apoptosis was observed in MARCH5^KO^ H1299 cells (see later in Fig. S9), indicating the effects were not specific to one cell line.

Conceptually, the stimulation of apoptosis in MARCH5^KO^ is paradoxical because stabilization of MCL1 is expected to inhibit apoptosis. One possibility is that MCL1 and MARCH5 can regulate mitotic apoptosis independently. To test this this hypothesis, we analyzed the effects of MCL1 expression on MARCH5^KO^-mediated apoptosis. Figure 3B shows that the mitotic apoptosis induced by MARCH5 KO could be circumvented by expressing more MCL1, suggesting that the accumulation of MCL1 after MARCH5 ablation should have an anti-apoptotic effect.

We next generated cell lines lacking both MARCH5 and MCL1 (Fig. 3C). As shown above, ablating MARCH5 or MCL1 individually promoted mitotic apoptosis. Significantly, ablating both MARCH5 and MCL1 together triggered more extensive apoptosis than individual component alone, suggesting that MCL1 did have a protective function on MARCH5^KO^-mediated apoptosis. This was further confirmed using live-cell imaging: KO of MARCH5 further accelerated mitotic apoptosis in an MCL1^KO^ background (Fig. 3D).

Collectively, these data indicate that although the accumulated MCL1 exerts an anti-apoptotic effect, it cannot overcome the mitotic apoptosis triggered by MARCH5 downregulation.

### MARCH5 regulates mitotic apoptosis independently on NOXA, BIM, and BID

**Fig. 4:**
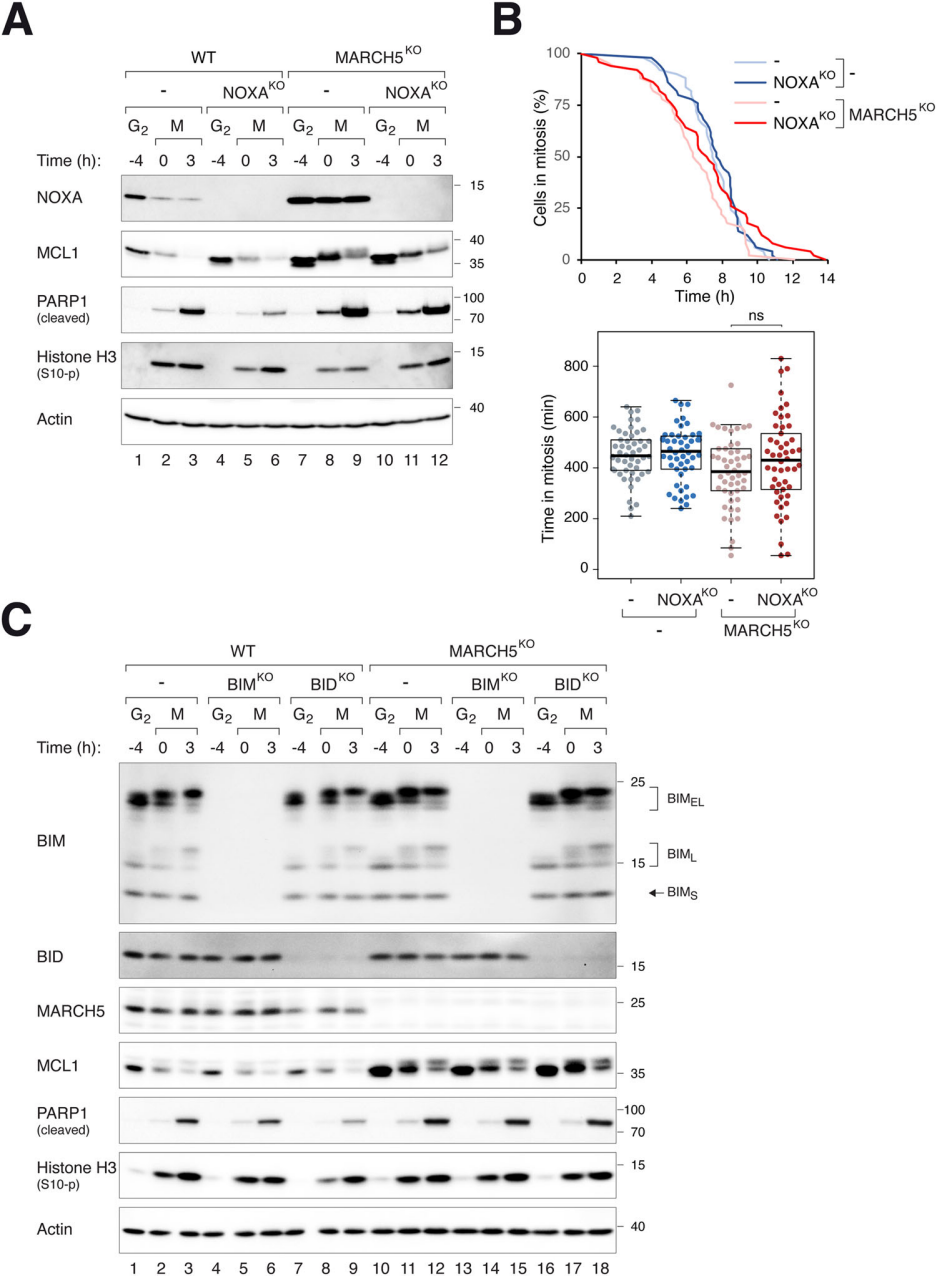
MARCH5 regulates mitotic apoptosis independently on NOXA, BIM, and BID. **A** KO of NOXA does not affect MARCH5^KO^-mediated mitotic apoptosis. NOXA^KO^ was generated in HeLa or MARCH5^KO^ backgrounds. The cells were synchronized and arrested in mitosis as before. Protein expression was analyzed with immunoblotting. **B** NOXA does not alter the duration of mitotic arrest in MARCH5^KO^. Control or NOXA^KO^ in MARCH5^KO^ cells (in a mAID-MCL1 and MCL1^KO^ background) were synchronized and arrested in mitosis as before. Individual cells were tracked using live-cell imaging. The duration of mitotic arrest is plotted using Kaplan–Meier estimator. Box-and-whisker plots show the elapsed time between mitotic entry and mitotic apoptosis/slippage. ns *p* > 0.05. **C** KO of BIM or BID does not affect MARCH5^KO^-mediated mitotic apoptosis. BIM^KO^ or BID^KO^ was generated in HeLa or MARCH5^KO^ backgrounds. The cells were synchronized and arrested in mitosis as before. Protein expression was analyzed with immunoblotting. The positions of splice variants of BIM (BIM_S_, BIM_L_, and BIM_EL_) are indicated.

NOXA and BIM are BH3-only proteins that bind and regulate the stability of MCL1 [30–32]. Degradation of MCL1 by MARCH5 is dependent on NOXA [23–26]. Similar to MCL1, NOXA is increased upon disruption of MARCH5 [26] (Fig. 4A; see also Fig. 3B, C). To determine if NOXA plays a role in MARCH5^KO^-mediated apoptosis, NOXA was further ablated in WT or MARCH5^KO^ cells. Figure 4A shows that KO of NOXA in WT cells stabilized interphase MCL1. Gene disruption of NOXA in MARCH5^KO^ cells, however, did not affect mitotic apoptosis (Fig. 4A, B). Similar results were obtained by disrupting NOXA and MARCH5 in H1299 cells (Fig. S8).

Similar experiments were performed by ablating BIM in either a WT or MARCH5^KO^ background. However, neither MCL1 expression nor PARP1 cleavage was affected by BIM disruption (Fig. 4C). Together with MCL1, BID is one of the few proteins that has been shown to be upregulated after disruption of MARCH5 [20]. Nevertheless, removal of BID did not affect mitotic apoptosis in MARCH5^KO^ cells (Fig. 4C).

These data show that BH3-only proteins that has been implicated in the MARCH5–MCL1 pathway (NOXA, BIM, and BID) do not influence MARCH5^KO^-mediated mitotic apoptosis.

### BAK is an executor of MARCH5^KO^-mediated mitotic apoptosis

**Fig. 5:**
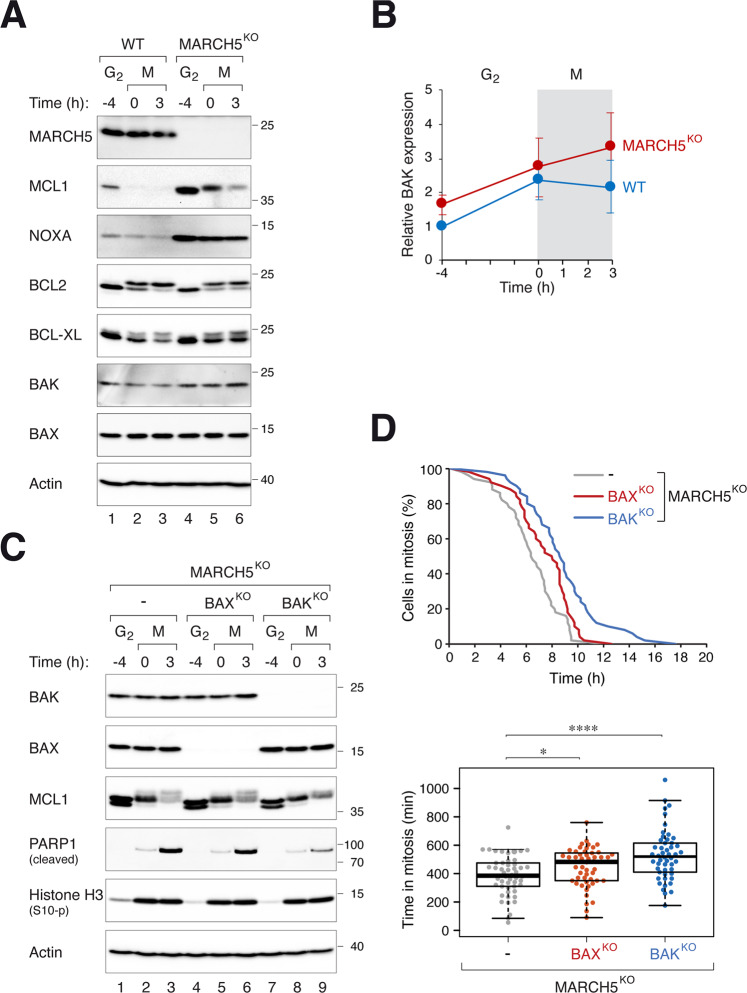
MARCH5 regulates mitotic apoptosis through BAK. **A** Disruption of MARCH5 elevates BAK expression. WT and MARCH5^KO^ cells were synchronized and arrested in mitosis as before. The expression of the indicated proteins was analyzed with immunoblotting. Note that several proteins (MCL1, BCL2, and BCL-XL) displayed mitotic gel mobility shifts. **B** Increased expression of BAK during mitotic arrest in MARCH5^KO^. WT or MARCH^KO^ cells were synchronized and arrested in mitosis as before. The expression of BAK during G_2_ and mitosis was quantified from immunoblots of three independent experiments (mean ± SEM). **C** PARP1 cleavage in MARCH^KO^-mediated mitotic apoptosis is reduced in the absence of BAK. BAX or BAK was ablated with CRISPR-Cas9 in MARCH5^KO^ cells. The cells were synchronized and arrested in mitosis as before. Protein expression was analyzed with immunoblotting. **D** MARCH^KO^-mediated mitotic apoptosis is delayed in the absence of BAK. MARCH5^KO^, MARCH5^KO^BAX^KO^, and MARCH5^KO^BAK^KO^ (all were MCL1^KO^ cells expressing ^mAID^MCL1) were transiently transfected with histone H2B-GFP before synchronized and arrested in mitosis as before. Individual cells were then tracked using live-cell imaging. The duration of mitotic arrest is plotted using Kaplan–Meier estimator. Box-and-whisker plots show the elapsed time between mitotic entry and mitotic apoptosis/slippage. *p < 0.05; ****p < 0.0001.

We found that although not as profound as MCL1, NOXA, and BID, the pro-apoptotic protein BAK was also marginally elevated in the absence of MARCH5 (Fig. 5A, B). To determine if pro-apoptotic proteins BAK and BAX contribute to MARCH5^KO^-mediated mitotic apoptosis, we generated MARCH5^KO^ cells lacking either BAK or BAX. Figure 5C shows that the loss of BAK in MARCH5^KO^ reduced PARP1 cleavage during mitotic arrest. The duration of mitotic arrest before apoptosis was also extended (Fig. 5D). By contrast, KO of BAK in a WT background did not affect the timing of mitotic apoptosis (Fig. S9A, B). The effect of BAX on MARCH5^KO^-dependent mitotic apoptosis was less significant than that of BAK in both PARP1 cleavage assay and single cell analysis (Fig. 5C, D). The dependency of MARCH5^KO^-mediated mitotic apoptosis on BAK was also found in H1299 cells (Fig. S9C), indicating that the effects were not specific for HeLa.

**Fig. 6:**
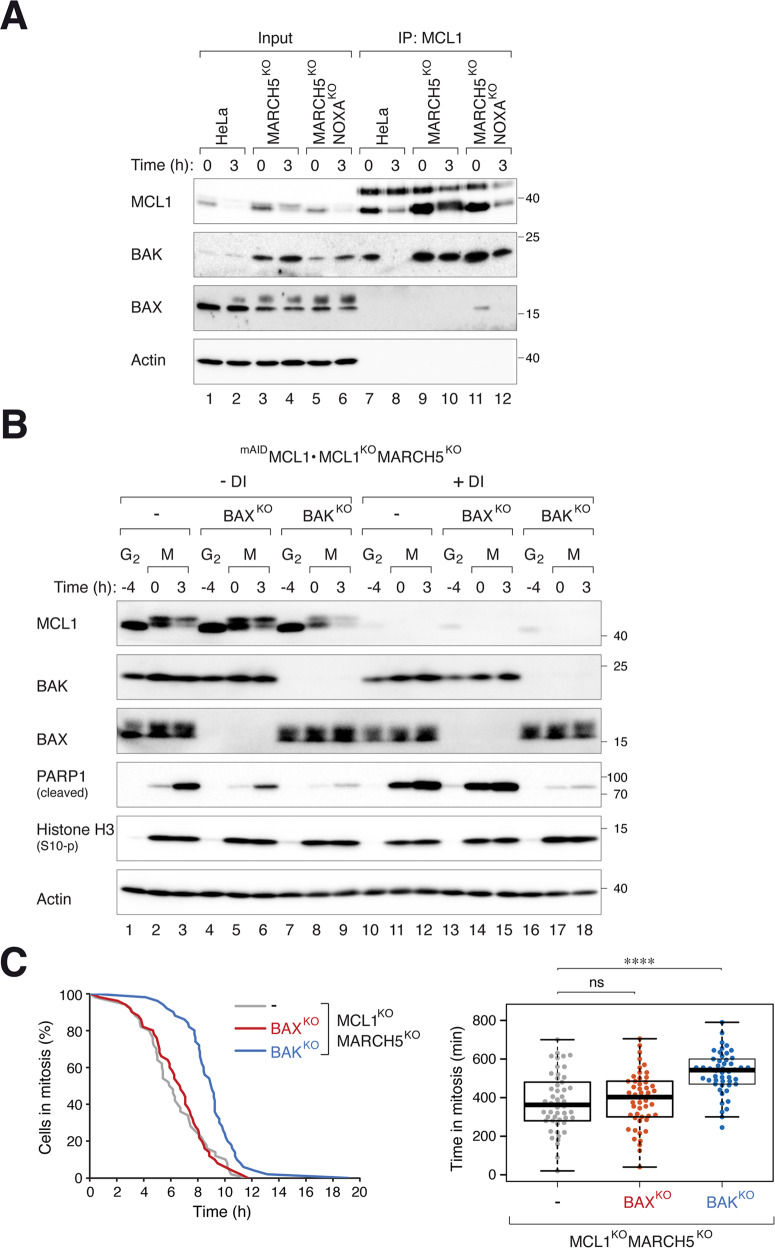
BAK-dependent mitotic apoptosis caused by MARCH5^KO^ does not require MCL1. **A** KO of MARCH5 promotes MCL1–BAK complex formation. HeLa, MARCH5^KO^, or MARCH5^KO^ NOXA^KO^ were synchronized and arrested in mitosis as before. Cell lysates prepared from different time points were subjected to immunoprecipitation using an anti-MCL1 antiserum. Both the total lysates and immunoprecipitates were analyzed with immunoblotting. **B** KO of BAK inhibited mitotic apoptosis in cells lacking MARCH5 and MCL1. BAX^KO^ or BAK^KO^ was generated from MARCH5^KO^ MCL1^KO^ cells expressing ^mAID^MCL1. The cells were synchronized and arrested in mitosis as before. ^mAID^MCL1 was turned off with DI at the time of second thymidine release. Protein expression was analyzed with immunoblotting. **C** Disruption of BAK delayed mitotic apoptosis in MARCH5^KO^ MCL1^KO^ cells. Cells were synchronized and treated as in **B** (except that they were transiently transfected with histone H2B-GFP before synchronization). The cells were incubated with DI to turn off ^mAID^MCL1 before individual cells were tracked using live-cell imaging. The duration of mitotic arrest is plotted using Kaplan–Meier estimator. Box-and-whisker plots show the elapsed time between mitotic entry and mitotic apoptosis/slippage. ****p < 0.0001; ns *p* > 0.05.

The amounts of MCL1–BAK complexes during mitosis were elevated in MARCH5^KO^ cells (Fig. 6A; decreased over time due to progressive MCL1 degradation). To test if MARCH5^KO^-mediated increase of MCL1 affects BAK-dependent mitotic apoptosis, we generated cells lacking BAX/BAK in a MARCH5^KO^ and MCL1^KO^ (rescued with ^mAID^MCL1) background. Mitotic apoptosis in MARCH5^KO^ cells in the presence of ^mAID^MCL1 was dependent on both BAK and BAX (Fig. 6B; -DI). The further increase in mitotic apoptosis after ^mAID^MCL1 was turned off was BAK- (but not BAX-) dependent (Fig. 6B). Consistent data were obtained using live-cell imaging, which indicated that KO of BAK delayed mitotic apoptosis in cells lacking both MARCH5 and MCL1 (Fig. 6C). We further showed that in the presence of MARCH5, mitotic apoptosis associated with MCL1^KO^ was only marginally affected by BAK or BAX (Fig. S10).

Taken together, these results demonstrated that in MARCH5^KO^ cells, while MCL1-regulated mitotic apoptosis acts through both BAK and BAX, MCL1-independent mitotic apoptosis only acts through BAK.

### NOXA contributes to mitotic apoptosis in MARCH5^KO^ by regulating MCL1–BAX complexes

**Fig. 7:**
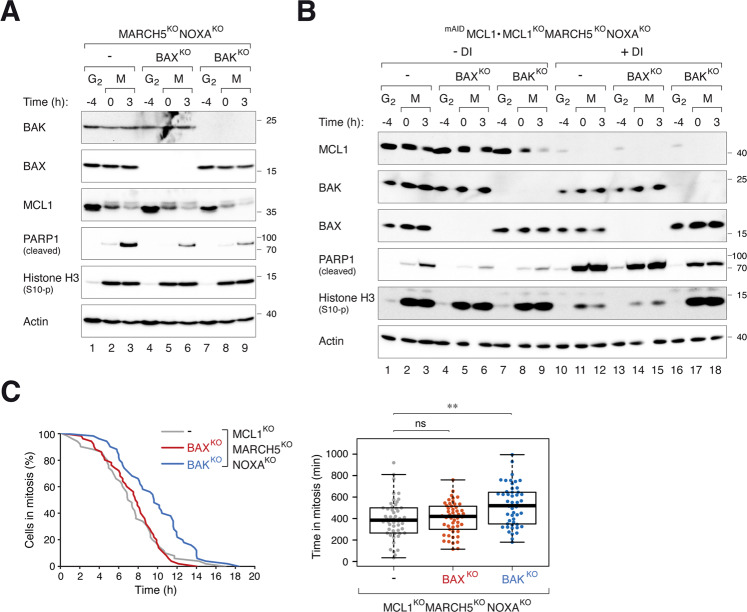
NOXA contributes to mitotic apoptosis by regulating MCL1–BAX. **A** Mitotic apoptosis in MARCH5^KO^ NOXA^KO^ is both BAX- and BAK-dependent. BAX^KO^ or BAK^KO^ was generated from MARCH5^KO^ NOXA^KO^ cells. The cells were synchronized and arrested in mitosis as before. Protein expression was analyzed with immunoblotting. **B** BAX-dependent mitotic apoptosis in MARCH5^KO^ NOXA^KO^ is mediated by MCL1. BAX^KO^ or BAK^KO^ was generated from MARCH5^KO^ NOXA^KO^ MCL1^KO^ cells expressing ^mAID^MCL1. The cells were synchronized and arrested in mitosis as before. ^mAID^MCL1 was turned off with DI at the time of second thymidine release. Protein expression was analyzed with immunoblotting. **C** Disruption of BAK delayed mitotic apoptosis in MARCH5^KO^ NOXA^KO^ MCL1^KO^ cells. Cells were synchronized and treated as in **B** (except that they were transiently transfected with histone H2B-GFP before synchronization). The cells were incubated with DI to turn off ^mAID^MCL1 before individual cells were tracked using live-cell imaging. The duration of mitotic arrest is plotted using Kaplan–Meier estimator. Box-and-whisker plots show the elapsed time between mitotic entry and mitotic apoptosis/slippage. **p < 0.01.

Unlike MCL1–BAK, MCL1–BAX was not detected in MARCH5^KO^ cells unless NOXA was also deleted (Fig. 6A). As NOXA was stabilized in MARCH5^KO 26^ (Fig. 4A), a possibility is that the accumulation of MCL1–NOXA prevented MCL1 from binding to BAX. In agreement with this hypothesis, MARCH5^KO^-mediated mitotic apoptosis became more dependent on BAX when NOXA was also deleted (Fig. 7A; compare to MARCH5^KO^ alone in Fig. 5C).

Also consistent with the hypothesis, the involvement of BAX for mitotic apoptosis required the presence of MCL1. Mitotic apoptosis in ^mAID^MCL1-expressing cells (in a MCL1^KO^ MARCH5^KO^ NOXA^KO^ background) was dependent on both BAX and BAK (Fig. 7B). After turning off ^mAID^MCL1, however, mitotic apoptosis became dependent on BAK only. These results were further verified using live-cell analysis (Fig. 7C).

Collectively, these data suggest a model in which MARCH5 disruption promotes BAK-dependent mitotic apoptosis. By contrast, BAX-dependent apoptosis Is suppressed by the accumulating NOXA (which in turn requires MCL1 accumulation).

### MARCH5 regulates mitotic apoptosis through DRP1

**Fig. 8:**
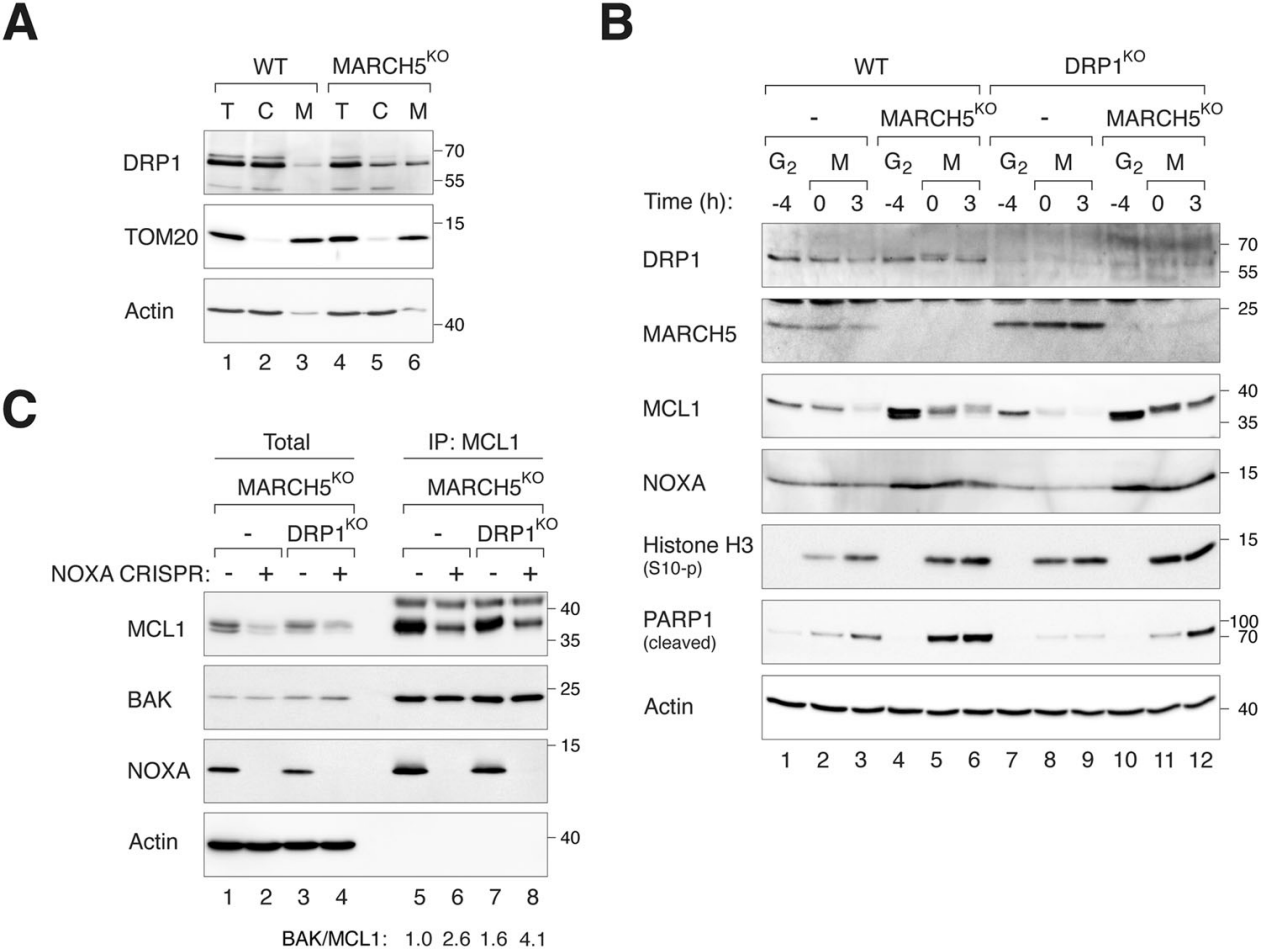
MARCH5^KO^-mediated mitotic apoptosis is dependent on DRP1. **A** MARCH5^KO^ increases DPR1 in mitochondrial fraction. Cellular fractionation of WT and MARCH5^KO^ cells were conducted to obtain lysates from total cell (T), cytosol (C), and mitochondrial-enriched heavy membrane (M) fractions. The expression of DRP1 was analyzed with immunoblotting. **B** Depletion of DRP1 alleviates mitotic apoptosis in MARCH5^KO^ cells. DRP1^KO^ cells were generated from HeLa WT or MARCH5^KO^ cells. The cells were synchronized and arrested in mitosis as before. Protein expression was analyzed by immunoblotting. **C** Depletion of DRP1 promotes MCL1–BAK interaction in NOXA-independent manner. NOXA^KO^ cells were generated from MARCH5^KO^ and DRP1^KO^MARCH5^KO^ cells. The cells were synchronized and arrested in mitosis as before. Mitotic cell lysates were subjected to immunoprecipitation using MCL1 antiserum. Both total and immunoprecipitates were analyzed by immunoblotting. The band intensities of MCL1 and BAK in the immunoprecipitates were quantified and the ratios of BAK/MCL are indicated.

MARCH5 regulates mitochondrial fission and fusion by interacting with and ubiquitinating the mitochondrial fission factor DRP1 and/or its receptor MiD49 on the mitochondrial outer membrane [19–21, 33, 34]. Consistent with previous results, depletion of MARCH5 did not alter total DRP1 expression but promoted its mitochondrial recruitment (Fig. 8A) as well as mitochondrial fission (Fig. S11A) during interphase. As expected, ablation of DRP1 abolished the mitochondrial fission induced in MARCH5^KO^ cells (Fig. S11A).

By contrast to interphase cells, the differences of mitochondrial fragmentation between WT and MARCH5^KO^ cells were less significant during mitotic arrest (Fig. S11B). Although further deletion of DRP1 reduced mitochondrial fragmentation in MARCH5^KO^ cells, the lack of effect of MARCH5 during mitotic arrest suggests that mitochondrial fragmentation per se may not play a significant role in MARCH5^KO^-mediated mitotic apoptosis. Importantly, depletion of DRP1 reduced PARP1 cleavage (Fig. 8B), indicating that mitochondrial accumulation of DRP1 is involved in MARCH5^KO^-induced mitotic apoptosis. The stability of mitotic MCL1 in MARCH5^KO^ cells was unaffected by DRP1. MCL1–BAK interaction was unchanged or marginally increased when DRP1 was deleted in MARCH5^KO^ cells (Fig. 8C). The increase was relatively minor comparing with the effect of MARCH5 on MCL1–BAK interaction (Fig. 6A).

Taken together, these results suggest that DRP1 promotes MARCH5^KO^-mediated mitotic apoptosis.

## Discussion

The importance of MCL1 in controlling the timing of apoptosis during mitotic arrest has been well-documented [9]. We confirmed in the cell model we used that mitotic apoptosis could be delayed or accelerated after ectopic expression of MCL1 (Fig. 3B) or KO of MCL1 (Figs. 3C and S4), respectively. Although MARCH5 is not solely responsible for the degradation of MCL1 during mitotic arrest, it is pivotal for setting the initial level of MCL1 at mitotic entry (Fig. 2A). This in turn determines the duration of the anti-apoptotic signals from MCL1 persists during mitotic arrest. In support of this, apoptosis was promoted after MCL1 was removed in both WT and MARCH5^KO^ background (Fig. 3C, D).

No clear consensus has been established regarding the precise mechanisms targeting MCL1 for degradation during mitotic arrest. The dependency of MCL1 degradation on the proteasome is generally accepted. For example, MCL1 could be stabilized by the proteasome inhibitor MG132 during mitosis (Figs. 1C and S7). Although several ubiquitin ligases that have been implicated in MCL1 degradation, including APC/C^CDC20^ (Figs. 1D and S1), SCF complexes (Fig. S1C), or MARCH5 (Fig. 2A), their inactivation did not prevent mitotic degradation of MCL1. Although KO of MARCH5 increased the stability of MCL1 (Fig. S3), MCL1 was still decreased during mitotic arrest. We cannot rule out the possibilities that there is redundancy between different ubiquitin ligases or a yet-unidentified ubiquitin ligase is involved in the instability of MCL1 during mitosis. Alternatively, it is possible MCL1 is degraded by a proteasome- but ubiquitin-independent mechanism.

**Fig. 9:**
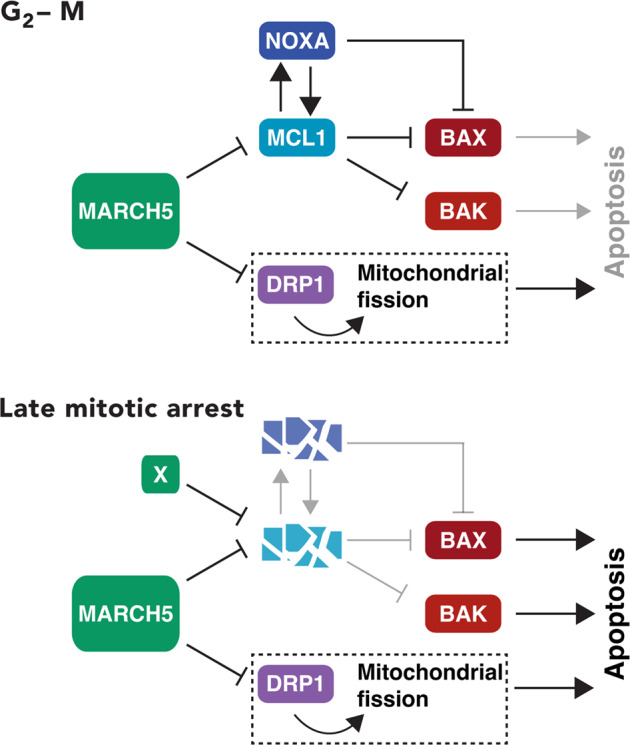
A model of the regulation of apoptosis during mitotic arrest by MARCH5. The ubiquitin ligase MARCH5 controls the stability of MCL1 during interphase. Mitotic degradation of MCL1 is proteasome-dependent but only partially dependent on MARCH5. Accordingly, MCL1 expression is elevated during early mitosis and takes longer to be depleted during mitotic arrest in MARCH5-depleted cells. NOXA is also stabilized in MARCH5-depleted cells in an MCL1-dependent manner. During late mitotic arrest, the destruction of MCL1 (by yet unidentified ubiquitin ligases X) and NOXA facilitates the activation of BAK and BAX. Our data suggest that the increase of mitochondrial DRP1 also plays a critical role in promoting mitotic apoptosis in the absence of MARCH5.

Although KO of MARCH5 increased the abundance of MCL1 during interphase, apoptosis was promoted during the subsequent mitotic arrest (Fig. 2A, B). The increase in mitotic apoptosis after MARCH5 ablation was independent on MCL1 (Fig. 3C, D). A solution to these paradoxical results is possible if we assume that another pro-apoptotic signal is stabilized or activated in the absence of MARCH5 (see Fig. 9 for a model). The increase of this pro-apoptotic signal was able to overcome the anti-apoptotic signals from the overall increase in MCL1. One candidate is the mitochondrial fission factor DRP1, which interacts with MARCH5 [19–21, 33, 34] and plays a role in apoptosis by interacting with BAX [35], thereby stimulating BAX oligomerization and cytochrome c release [36]. However, it has been shown that siRNAs against DRP1 promotes mitotic cell death [37]. We found that disruption of DRP1 decreased mitotic apoptosis in MARCH5^KO^ cells (Fig. 8B), suggesting that DRP1 pathway could account for the increase of mitotic apoptosis in MARCH5^KO^ cells. As mitochondrial fission during mitosis was not significantly altered (Fig. S11), it is currently unclear if mitochondrial fission per se is responsible for the elevated mitotic apoptosis in MARCH5^KO^ cells.

MCL1 is degraded when it forms a complex with NOXA [24, 30, 31, 38]. In agreement, NOXA KO enhanced MCL1 stability in interphase (Fig. 4A). Although NOXA KO did not prevent mitotic degradation of MCL1, the accumulation of MCL1 during early mitosis probably explains the reduction of mitotic apoptosis (Fig. 4A).

In agreement with recently published data [26], we found that NOXA was stabilized in the absence of MARCH5 (Fig. 2A). This stabilization of NOXA was dependent on the presence of MCL1 (Fig. 3C). The regulation was not reciprocal, as the stabilization of MCL1 in MARCH5^KO^ was not dependent on NOXA (Fig. 4A). This indicated that the regulation of MCL1 by MARCH5 was more pronounced than that by NOXA. Similarly, the rate of mitotic apoptosis in MARCH5^KO^ was unaffected by NOXA (Fig. 4A, B). These results differ from the conclusion from Haschka et al. [26], in which they showed that knockdown of NOXA with siRNA abolished cell death in MARCH5-disrupted cells.

In addition to MCL1 and NOXA, BIM and BID have also been implicated to be under MACRH5’s regulation. However, KO of BIM or BID did not affect the rate of MARCH5^KO^-mediated mitotic apoptosis (Fig. 4). Although BIM itself can promote mitotic apoptosis [9], KO of BIM did not affect apoptosis associated with MARCH5^KO^ (Fig. 4C).

By contrast, KO of the pro-apoptotic protein BAK (but not the related BAX) delayed mitotic apoptosis in MARCH5^KO^, indicating MARCH5 deletion promoted BAK-dependent apoptosis (Fig. 5C, D). Interestingly, KO of BAK or BAX alone (in MARCH5-containing background) did not affect mitotic apoptosis (Fig. S9). Although an accumulation of BAK after MARCH5 disruption was observed repeatedly, the magnitude of change was small compared to that of MCL1 or NOXA (Fig. 5A, B). Hence it is unlikely that BAK enrichment is the sole explanation for the increase in apoptosis after MARCH5 disruption. We postulate that another target of MARCH5 was responsible for triggering mitotic apoptosis in MARCH5^KO^ cells. As MARCH5 regulates several proteins involved in mitochondrial physiology including fission and fusion (see introduction), a tantalizing speculation is that mitotic apoptosis may involve dysregulating mitochondrial functions (Fig. 9).

Mitotic apoptosis associated with the loss of MCL1 was also reduced in the absence of BAK (Fig. S10). The lack of contribution of BAX to MARCH5^KO^-mediated mitotic apoptosis could be due to the accumulation of NOXA, as apoptosis became BAX-dependent after KO of NOXA in MARCH5^KO^ cells but not MARCH5^KO^ MCL1^KO^ double KO cells (Fig. 7).

An obvious implication of the functions of MARCH5 and MCL1 in mitotic apoptosis is their effects on tumorigenesis and responses to anti-mitotic drugs. MCL1 is one of the most frequently dysregulated apoptotic genes in cancers. In a large scale cancer genome study, MCL1 was found to be one of the most frequently amplified genes in cancers [39]. Elevated levels of MCL1 contribute to resistance to both conventional chemotherapies and targeted therapies such as the BCL2 inhibitor Venetoclax [40]. MARCH5 is also altered in a selected group of cancers. For example, deletion of MARCH5 was detected in up to 5% of pancreatic cancer and is associated with shorter progression-free survival [25]. Downregulation of MARCH5 is predicted to sensitize cells to antimitotic treatments. As MARCH5 can regulate mitotic apoptosis in a MCL1-independent mechanism, it is possible that targeting MARCH5 can promote sensitivity to antimitotic treatments even in cancer cells overexpressing MCL1.

## Methods

### Plasmids

CRISPR-Cas9 plasmids were generated by annealing the indicated pairs of oligonucleotides followed by ligation into BbsI-cut pX330 (obtained from Addgene, Cambridge, MA, USA; #42230): APC4 (5′‑CACCGTTAAGCTCTTGGGAGACGTC‑3′ and 5′‑AAACGACGTCTCCCAAGAGCTTAAC‑3′); BAK (5′‑CACCGACGGCAGCTCGCCATCATCG‑3′ and 5′‑AAACCGATGATGGCGAGCTGCCGTC‑3′); BAX (5′‑CACCGCTGCAGGATGATTGCCGCCG‑3′ and 5′‑AAACCGGCGGCAATCATCCTGCAGC‑3′);); DRP1 (5′‑CACCGCTGCCTCAAATCGTCGTAG‑3′ and 5′‑AAACCTACGACGATTTGAGGCAGC‑3′); MARCH5 (5′‑CACCGGCCTGTCTACAACGCTGGG‑3′ and 5′‑AAACCCCAGCGTTGTAGACAGGCC‑3′); MCL1 (5′‑CACCGCTCAAAAGAAACGCGGTAAT‑3′ and 5′‑AAACATTACCGCGTTTCTTTTGAGC‑3′); NOXA (5′‑CACCGACGCTCAACCGAGCCCCGCG-3′ and 5′‑AAACCGCGGGGCTCGGTTGAGCGTC-3′); and SKP1 (5′‑CACCGTGTTGTTGTAGGTCATTCAG-3′ and 5′‑AAACCTGAATGACCTACAACAACAC-3′).

MCL1 in pcDNA3.1/V5-His-TOPO was obtained from Addgene (#21605). The MCL1 cDNA was amplified with PCR (primers 5′‑GAGAATTCATGTTTGGCCTCAAAAGAAACGCGGTA‑3′ and 5′‑CAGGATCCCTATCTTATTAGATATGCCAAACCAGC‑3′); the PCR product was then cut with EcoRI and BamHI and ligated into pUHD-P3 [41] to generate FLAG-MCL1 in pUHD-P3. To generate a CRISPR-resistant MCL1, the primers 5′‑GAGAATTCATGTTTGGCCTGAAGAGGAATGCG‑3′ and 5′‑ACGGATCCCTATCTTATTAGATATGCCAAACCAGC‑3′ were used to introduce silent mutations by PCR using FLAG-MCL1 in pUHD-P3 as a template; the PCR products were cut with EcoRI and BamHI and ligated into EcoRI- and BamHI-cut pUHD-SB-mAID [28] to generate mAID-MCL1 in pUHD-SB-mAID. FLAG-MCL1 in pUHD-P3 was amplified by PCR using 5′‑ACCCCAAGCTGGCCTCTGCGGGCTA‑3′ and 5′‑CGAGCTCTAGAGAATTGATCATGTCTGG‑3′; the PCR product was inserted to NcoI-HindIII-cut pSBbi-TIR1/Bla [42] using a Seamless Ligation Cloning Extract (SLiCE) cloning method [43] to generate FLAG-MCL1 in pSBbi/Bla (a plasmid that constitutively expressed ^FLAG^MCL1).

CRISPR-resistant mAID-APC4 and TIR1-myc in pSBbi-Pur were generated as previously described [6]. Sleeping Beauty transposase pCMV(CAT)T7-SB100 was obtained from Addgene (#34879). FLAG-cyclin A in pUHD-P1 was generated as previously described [44]. To obtain FLAG-SKP1 in pUHD-SB/Hyg, SKP1 in pUHD-P1 [45] was amplified by PCR (primers: 5′‑AGCTCGTTTAGTGAACCGTCAGATCG‑3′ and 5′‑GCGGATCCTCACTTCTCTTCACACC‑3′; the PCR product was cut with NcoI and BamH1 and ligated to NcoI- and BamH1-cut pUHD-SB/Hyg [28].

### Cell lines

HeLa (cervical carcinoma) used in this study was a clone expressing the tTA tetracycline transactivator [44]. H1299 cells were obtained from American Type Culture Collection (Manassas, VA, USA). KO cells were generated by transfecting cells with specific CRISPR-Cas9 plasmids and a plasmid expressing blasticidin-resistant gene (a gift from Tim Hunt, Cancer Research UK). Transfected cells were enriched by culturing cells with blasticidin-containing medium for 36 h before seeded onto 10-cm plates (for isolation of mixed population) or 96-well plates (for isolation of single cell-derived colonies). MARCH5^KO^ cells were generated by transfecting HeLa cells with MARCH5 CRISPR-Cas9 in pX330. Single cell-derived MARCH5^KO^ colonies were isolated for experiments. The MARCH5^KO^ cells were further transfected with NOXA CRISPR-Cas9 to generate MARCH5^KO^NOXA^KO^ double KO cells. The cells were transfected with CRISPR-Cas9 against BAK or BAX and selected for generating MARCH5^KO^NOXA^KO^BAK^KO^ or MARCH5^KO^NOXA^KO^BAX^KO^ triple KO cells, respectively. DRP1^KO^ cells were obtained by transfecting HeLa cells with DRP1 CRISPR-Cas9 in pX330. Single cell-derived DRP1^KO^ cells colonies were isolated for experiments. The stable DRP1^KO^ cells were further transfected with MARCH5 and/or NOXA CRISPR-Cas9 to generate DRP1^KO^MARCH5^KO^ double KO cells and DRP1^KO^MARCH5^KO^NOXA^KO^ triple KO cells. APC4^KO^ cells expressing ^mAID^APC4 were as previously described [6]. This cell line was then transfected with MARCH5 CRISPR-Cas9 for isolating individual colonies. MCL1^KO^ cells expressing ^mAID^MCL1 were generated by transfecting HeLa with MCL1 CRISPR-Cas9, mAID-MCL1 in pUHD-SB-mAID/Hyg, pSBbi-TIR1/Pur and pCMV(CAT)T7-SB100. Transfected cells were selected by culturing in hygromycin- and puromycin-containing medium for 7 days. Single cell-derived colonies were isolated by seeding at low density in 96-well plates. The cells were further transfected with MARCH5 CRISPR-Cas9 to isolate single cell-derived colonies of MARCH5^KO^MCL1^KO^ cells expressing ^mAID^MCL1. This cell line was transfected with NOXA CRISPR-Cas9 plasmid to generate NOXA^KO^MARCH5^KO^MCL1^KO^ cells expressing ^mAID^MCL1. Both MARCH5^KO^MCL1^KO^ and NOXA^KO^MARCH5^KO^MCL1^KO^ expressing ^mAID^MCL1 were transfected with CRISPR-Cas9 against either BAK or BAX to further KO BAK or BAX, respectively. ^FLAG^MCL1 was overexpressed in HeLa or MARCH5^KO^ cells by transfecting the respective cell lines with FLAG-MCL1 in pSBbi/Bla and pCMV(CAT)T7-SB100. BAK^KO^, BAX^KO^, or NOXA^KO^ cells were obtained by transfecting HeLa cells with CRISPR-Cas9 plasmids against BAK, BAX, or NOXA respectively. CDC20^KO^ cells expressing ^HA^CDC20 were generated as previously described [6]. To generate SKP1^KO^ cells expressing ^FLAG^SKP1, HeLa cells were transfected with SKP1 CRISPR-Cas9, FLAG-SKP1 in pUHD-SB/Hyg, and pCMV(CAT)T7-SB100. The cells were cultured with hygromycin-containing medium for 7 days before seeded onto 96-well plates at low density to obtain single cell-derived colonies. ^FLAG^BCL-XL-expressing HeLa cells were generated as previously described [46]. This cell line was further transfected with MCL1 CRISPR-Cas9 and seeded onto 96-well plates at low density to isolate MCL1^KO^ cells expressing ^FLAG^BCL-XL. MARCH5^KO^ in H1299 cells was generated by transfecting H1299 with MARCH5 CRISPR-Cas9 in pX330. The cells were further transfected with plasmids expressing NOXA or BAK CRISPR-Cas9 to obtain MARCH5^KO^NOXA^KO^ or MARCH5^KO^BAK^KO^ double KO cell lines, respectively.

### Sequencing and indel analysis

Purified genomic DNA from MARCH5^KO^ cells were amplified with PCR using primers 5′-TGGTTCATTAGAGAAGGTGAAGAATTACTG-3′ and 5′-TGTTATTCAATTATATCCACACAGGTATGC-3′, which covers MARCH5 CRISPR-Cas9 edited locus. PCR products were sequenced using primer 5′-TGGTTCATTAGAGAAGGTGAAGAATTACTG-3′. Indel analysis was conducted using ICE Analysis Tool (Synthego Performance Analysis, ICE Analysis. 2019. v2.0).

### Cell culture

Cells were propagated in Dulbecco’s modified Eagle’s medium (DMEM) supplemented with 10% (v/v) calf serum (for HeLa) or fetal bovine serum (for H1299) and 50 U/ml of penicillin streptomycin (Thermo Fisher Scientific, Waltham, MA, USA). Cells were treated with the following reagents at the indicated final concentration: blasticidin S HCl (Gibco, Thermo Fisher Scientific, Waltham, MA, USA; 3.75 µg/ml), doxycycline (Dox) (2 µg/ml), hygromycin B (Invitrogen, Carlsbad, CA, USA; 250 µg/ml), indole-3-acetic acid (Sigma-Aldrich; 50 µg/ml), Z-Leu-leu-leu-al (MG132) (Selleckchem, Houston, TX, USA; 10 µM), nocodazole (NOC) (Sigma-Aldrich, St. Louis, MO, USA; 100 ng/ml), puromycin (Sigma-Aldrich, St. Louis, MO, USA; 300 ng/ml), RO3306 (Selleckchem; 10 µM), cycloheximide (CHX) (Sigma-Aldrich, St. Louis, MO, USA; 10 µg/ml), and thymidine (Acros Organics, Fair Lawn, NJ, USA; mM). Cells were transfected using a calcium phosphate precipitation method [47]. Synchronization with double thymidine and NOC shake-off were as previously described [48]. The cells were washed with PBS at 8 h after release from the second thymidine block. The attached cells were then harvested as G_2_ samples. A portion of the G_2_ cells were incubated with NOC for another 4 h. Mitotic cells were isolated with mechanical shake-off and further incubated with NOC for different time points or released from NOC for 2 h as G_1_ samples.

### Live-cell imaging

Cells were seeded onto 12-well or 24-well cell culture plates and placed into an automated microscopy system with temperature, humidity, and CO_2_ control chamber (Zeiss Celldiscoverer 7, Oberkochen, Germany). Images were captured every 5 or 10 min for 24 h. Data acquisition was carried out with Zeiss ZEN 2.3 (blue edition) and analysis was performed using ImageJ (National Institutes of Health, Bethesda, MD, USA). After mitosis, one of the daughter cells was randomly selected and continued to be tracked.

### Antibodies and immunological methods

The following antibodies were obtained from the indicated sources: monoclonal antibodies against beta-actin (Sigma-Aldrich), BAK (sc-517390, Santa Cruz Biotechnology), BCL-XL (A5091, Bimake), BIM (2933S, Cell Signaling Technology), cyclin A2 (AT10, a gift from Tim Hunt, Cancer Research UK), cyclin B1 (sc-245, Santa Cruz Biotechnology), cyclin E1 (HE12, Santa Cruz Biotechnology), DRP1 (sc-271583, Santa Cruz Biotechnology), NOXA (200-301-H98, Rockland), cleaved PARP1 (552597, BD Biosciences), SKP1 (H-6, Santa Cruz Biotechnology), TOM20 (sc-17764, Santa Cruz Biotechnology); polyclonal antibodies against APC4 (ab72149, Abcam), BAX (sc-493, Santa Cruz), BID (2002S, Cell Signaling Technology), phosphorylated histone H3^Ser10^ (sc-8656R, Santa Cruz Biotechnology), MARCH5 (19168S, Cell Signaling Technology), and MCL1 (sc-819, Santa Cruz Biotechnology). Immunoprecipitation was performed as previously described [6]. Polyclonal antibodies against MCL1 (sc-819, Santa Cruz Biotechnology) were used for immunoprecipitation of endogenous MCL1. Anti-FLAG (M2) Affinity Gel (A2220, Sigma-Aldrich) was used for immunoprecipitation of FLAG-tagged proteins. Immunoblotting was performed as previously described [49]. The positions of molecular size standards (in kDa) are indicated in the Figures. Quantification of signals on immunoblotting was conducted with Image Lab software (version 5.2.1 build 11, Bio-Rad Laboratories, Hercules, CA, USA). Uncropped Western blot images are shown in Fig. S12.

### Immunostaining

Mitotic cells were collected by mechanical shake off after exposing cells to NOC for 3 h. The cell suspension was added to 24-well plates containing poly-L-lysine (50 µg/ml) coated coverslips. After centrifugating the cells onto the coverslips (10 g for 2 min), the coverslips were washed with PBS and fixed with 4% formaldehyde in PBST (0.1% Tween 20 in PBS) at 25 °C for 10 min. The cells were washed with PBS (three times, 5 min each; same for other PBS/PBST washes) followed by blocking with 4% BSA in PBST for 1 h at 25 °C. The cells were then washed with PBST and incubated with TOM20 antibody (1:200) at 4 °C overnight. After washing with PBST, the cells were incubated with autoantibody against human ANA-centromere CREST (Fitzgerald Industries, Acton, MA, USA; 1:1000) at 25 °C for 2 h, followed by washing with PBST and incubating with Alexa-Flour-647 goat anti-human IgG and Alexa-Flour-488 goat anti-mouse IgG (Life Technologies; 1:1000) at 25 °C for 2 h. The coverslips were then incubated with Hoechst 33342 (Life Technologies; 1:25,000) at 25 °C for 15 min, wash with PBST, before mounted with 2% N-propyl-gallate (Sigma-Aldrich) in glycerol. Interphase cells were seeded on poly-L-lysin coated coverslips 24 h before fixation and followed the same immunostaining procedure as mitotic cells. Images were taken with Zeiss Celldiscoverer 7 (Zeiss, Oberkochen, Germany) before processed with Zeiss Zen 2.3 (blue edition) and ImageJ (National Institutes of Health, Bethesda, MD, USA). Mitochondrial morphology analysis (average mitochondrial network area, perimeter, aspect ratio, and form factor per cell) was conducted using ImageJ with the Mitochondria Analyzer plugin [50].

### Mitochondrial fractionation

Cells from 10-cm culture dishes at confluency were collected by centrifugation and resuspended in fractionation buffer containing 20 mM HEPES (pH 7.4), 1 mM EDTA, 1 mM EGTA, 10 mM KCl, and a protease inhibitor mix. The cell suspension was passed through a 27-gauge needle 15 times for homogenization. After centrifugated at 800 × *g* for 10 min, the supernatant was collected and further centrifugated at 10,000 × *g* for 10 min. The supernatant was collected as the cytosolic fraction and the pellet as mitochondria-enriched fraction.

### Statistical analysis

Box-and-whisker plots (center lines show the medians; box limits indicate interquartile range; whiskers extend to the most extreme data points that were no more than 1.5 times the interquartile range from the 25th and 75th percentiles) were generated using RStudio (version 1.2.5019; Boston, MA, USA). Mann-Whitney-Wilcoxon test was used to calculate statistical significance.

## Supplementary information

Supplemental Figure legends

Supplemental Figure S1

Supplemental Figure S2

Supplemental Figure S3

Supplemental Figure S4

Supplemental Figure S5

Supplemental Figure S6

Supplemental Figure S7

Supplemental Figure S8

Supplemental Figure S9

Supplemental Figure S10

Supplemental Figure S11

Supplemental Figure S12

Reproducibility Checklist
