# Supplementary material for: MARCH5 regulates mitotic apoptosis through MCL1-dependent and independent mechanisms
Source: Cell Death Differ. 2022 Nov 3;30(3):753–65. doi: 10.1038/s41418-022-01080-2 (PMC9984497; doi:10.1038/s41418-022-01080-2)
Supplement: Supplementary file 1 — Supplemental Figure legends [file 41418_2022_1080_MOESM1_ESM.pdf]

SUPPLEMENTAL FIGURE LEGENDS

Figure S1. Mitotic degradation of MCL1 is CDC20- and SKP1-independent.

(A) Depletion of CDC20 does not affect MCL1 stability. CDC20^{KO} cells expressing ^{HA}CDC20 were synchronized as described in Fig 1A with minor modifications: Dox was added to turn off the expression of ^{HA}CDC20 at 14 h after first thymidine release. Lysates were prepared and the indicated proteins were detected with immunoblotting. Note that the same samples and control panels (CDC20, phosphor-histone H3^{Ser10}, cyclin B1, and actin) published previously were used to analyze the expression of cyclin A and MCL1 [6]. The asterisk indicates the position of a cross-reactive band by the CDC20 antibodies.

(B) Cyclin A but not MCL1 interacts with CDC20 during mitosis. HeLa cells were transfected with either FLAG-tagged cyclin A or MCL1. The cells were synchronized in mitosis as described in Fig 1A. Lysates were prepared and subjected to immunoprecipitation using anti-FLAG beads. Both the total lysates and immunoprecipitates were analysed with immunoblotting.

(C) Mitotic degradation of MCL1 is SKP1-independent. HeLa or SKP1^{KO} cells expressing ^{FLAG}SKP1 were synchronized as described in Fig 1A: Dox was added to turn off the expression of ^{FLAG}SKP1 at 24 h before thymidine block. MG132 was applied at t=0 as indicated. Lysates were prepared and analysed with immunoblotting.

Figure S2. Indel analysis of MARCH5^{KO} cells.

MARCH5^{KO} cells were subjected to sequencing of the *MARCHF5* locus and indel analysis as described in Materials and Methods. **(A)** Sequencing trace of control (HeLa) and the edited samples (MARCH5^{KO}) were generated by ICE software for indel analysis. Targeted sequence of gRNA (solid black line),

PAM sequence (dotted red line), and edited site (dotted black line) are indicated. **(B)** Discordance calculated by ICE for edited (green) and control (grey) trace files. The alignment window (blue horizontal line) indicates the region of the traces with high Phred quality scores and is used for aligning the control and edited traces. The inference window (red horizontal line) indicates the altered sequences around the edited site (dotted black line). ICE-calculated discordance showed robust trace irregularity for approximating the location of editing. **(C)** Indel and corresponding prevalence calculated by ICE (R^2=0.98, editing efficiency = 98).

Figure S3. Depletion of MARCH5 increases the half-life of MCL1 during mitotic arrest.

WT and MARCH5^{KO} HeLa cells were synchronized by double-thymidine methods and arrested in mitosis as before. Mitotic cells were exposed to CHX or buffer and harvested at different time points for immunoblotting analysis. The MCL1 band intensity was quantified. The degradation rate constant k of WT or MARCH5^{KO} was calculated by linear regression of the intensities from first three time points (WT) or all time points (MARCH5). The half-life $T_{1/2}$ was calculated by the equation $T_{1/2} = ln(2)/k$.

Figure S4. Mitotic apoptosis is negatively regulated by MCL1.

(A) Generation of MCL1^{KO} in ^{FLAG}BCL-XL-overexpressing cells. HeLa cells overexpressing ^{FLAG}BCL-XL was first generated before MCL1 was disrupted with CRISPR-Cas9. The cells were incubated with Dox (to turn off ^{FLAG}BCL-XL) for 8 days. After incubation with NOC for 16 h, the cells were harvested and analyzed with immunoblotting. Note that due to the overexpression of exogenous ^{FLAG}BCL-XL, the endogenous BCL-XL cannot be seen in this exposure.

(B) Mitotic apoptosis is exacerbated in MCL1^{KO} cells. Cells expressing ^{FLAG}BCL-XL (with or without MCL1^{KO}) were treated with Dox for 7 days before the experiment to turn off the expression of ^{FLAG}BCL-XL. The cells were synchronized and arrested in mitosis as before. Protein expression was analyzed with immunoblotting. Note that the remaining BCL-XL signals after Dox treatment corresponds to the endogenous BCL-XL.

(C) Acceleration of mitotic apoptosis in MCL1^{KO} cells. Cells were synchronized as in panel B. At 4 h after release from the second thymidine block, individual cells were tracked using live-cell imaging. Key: interphase (grey); mitosis (red); truncated bars (apoptosis). The duration of mitotic arrest is plotted using Kaplan-Meier estimator. Box-and-whisker plots show the elapsed time between mitotic entry and mitotic apoptosis/slippage. ****P<0.0001.

Figure S5. NOC-mediated apptosis depends on mitotic arrest.

HeLa cells were synchronized using a double thymidine procedure and trapped in mitosis with NOC as described in Fig 1A. G_2 cells (t=-4 h) were incubated with either NOC (to isolate mitotic cells as before) or RO3306 (with or without NOC) (to trap cells continuously in G_2). A portion of NOC-blocked mitotic cells were also treated with RO3306 at t=0 to induce mitotic slippage. At different time points, lysates were prepared, and the indicated proteins were detected with immunoblotting.

Figure S6. Accelerated mitotic apoptosis in the absence of MCL1.

HeLa and MCL1^{KO} expressing ^{mAID}MCL1 were transiently transfected with histone H2B-GFP before synchronized and arrested in mitosis as before. The cells were either untreated or incubated with DI before individual cells were tracked using live-cell imaging for 24 h (starting at 8 h after second thymidine

release) (*n*=50). Key: interphase (grey); mitosis (red); interphase after mitotic slippage (blue); truncated bars (apoptosis).

Figure S7. Mitotic apoptosis is inhibited by proteasome inhibitors and correlates with MCL1 expression.

MCL1^{KO} cells expressing ^{mAID}MCL1 were synchronized and arrested in mitosis as before. ^{mAID}MCL1 was turned off with DI at the time of second thymidine release. Either buffer or MG132 was added after mitotic cells were isolated with mechanical shake-off (t=0). The expression of different proteins was analyzed with immunoblotting.

Figure S8. KO of MARCH5 increases MCL1 and apoptosis during mitotic arrest independently on NOXA in H1299 cells.

Parental H1299 (WT) and MARCH5^{KO} in the presence of absence of NOXA (NOXA^{KO}) were synchronized and arrested in mitosis as before. Protein expression was analyzed with immunoblotting.

Figure S9. KO of BAX or BAK alone does not affect mitotic apoptosis.

(A) PARP1 cleavage in mitotic apoptosis is not affected in the absence of BAX or BAK. BAX or BAK was ablated in HeLa cells. The cells were synchronized and arrested in mitosis as before. The expression of different proteins was analyzed with immunoblotting.

(B) The rate of mitotic apoptosis is not affected by BAX or BAK. HeLa, BAX^{KO}, or BAK^{KO} (all were MCL1^{KO} cells expressing ^{mAID}MCL1) were transiently transfected with histone H2B-GFP before synchronized and arrested in mitosis as before. Individual cells were then tracked using live-cell imaging. The duration of mitotic arrest is plotted using Kaplan-Meier estimator. Box-

and-whisker plots show the elapsed time between mitotic entry and mitotic apoptosis/slippage. ns $P>0.05$.

(C) MARCH5^{KO} promotes mitotic apoptosis in a BAK-dependent manner in H1299. MARCH5^{KO} were generated in H1299 cells. BAK was further disrupted in the MARCH5^{KO} to generate double-KO cells. WT H1299 and the two KO cell lines were synchronized using a double thymidine procedure and trapped in mitosis with NOC as before. The expression of different proteins was analyzed with immunoblotting.

Figure S10. KO of BAX or BAK only marginally affect mitotic apoptosis in MCL1^{KO} cells.

BAX^{KO} or BAK^{KO} was generated from MCL1^{KO} expressing ^{mAID}MCL1. The cells were synchronized and arrested in mitosis as before. ^{mAID}MCL1 was turned off with DI at the time of second thymidine release. The expression of different proteins was analyzed with immunoblotting.

Figure S11. DRP1 is involved in MARCH5^{KO}-mediated mitochondrial fission.

(A) Disruption of DRP1 abolishes MARCH5^{KO}-mediated mitochondrial fission in interphase. Asynchronous cells were seeded on coverslips for immunostaining. Z-projection of stack of 15 images (interval: 0.5 μm) per mitotic cells from WT, MARCH5^{KO}, DRP1^{KO}, and DRP1^{KO}MARCH5^{KO} immunostained for the mitochondrial marker TOM20 (red) and DNA (blue) are shown on the left (scale bar: 10 μm). The averages of area, perimeter, aspect ratio, and form factor per mitotic cell were quantified (n=20).

(B) Depletion of MARCH5 does not significantly affect mitochondrial fragmentation during mitotic arrest. Mitotic cells were collected by mechanical shake off after incubated in NOC-containing medium for 3 h and seeded on coverslips for immunostaining. Z-projection of stack of 15 images

(interval: 0.5 μm) per mitotic cells from WT, $MARCH5^{KO}$, $DRP1^{KO}$, and $DRP1^{KO}MARCH5^{KO}$ immunostained for the mitochondrial marker TOM20 (red) and DNA (blue) are shown on the left (scale bar: 5 μm). The averages of area, perimeter, aspect ratio, and form factor per mitotic cell were quantified (n=20).
