# Supplementary figures and images for: MARCH5 regulates mitotic apoptosis through MCL1-dependent and independent mechanisms
Source: Cell Death Differ. 2022 Nov 3;30(3):753–65. doi: 10.1038/s41418-022-01080-2 (PMC9984497; doi:10.1038/s41418-022-01080-2)

**A**

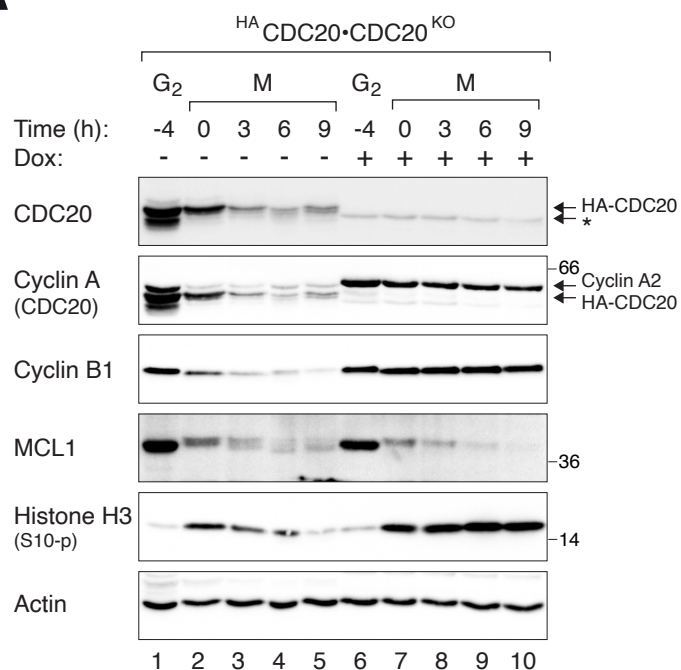

**B**

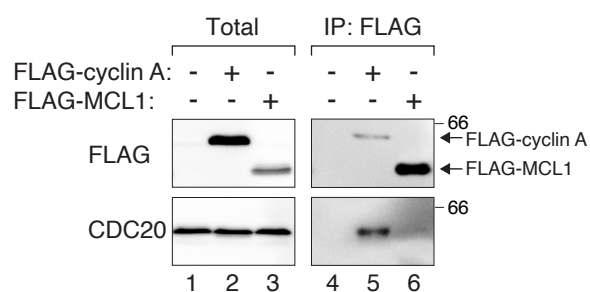

**C**

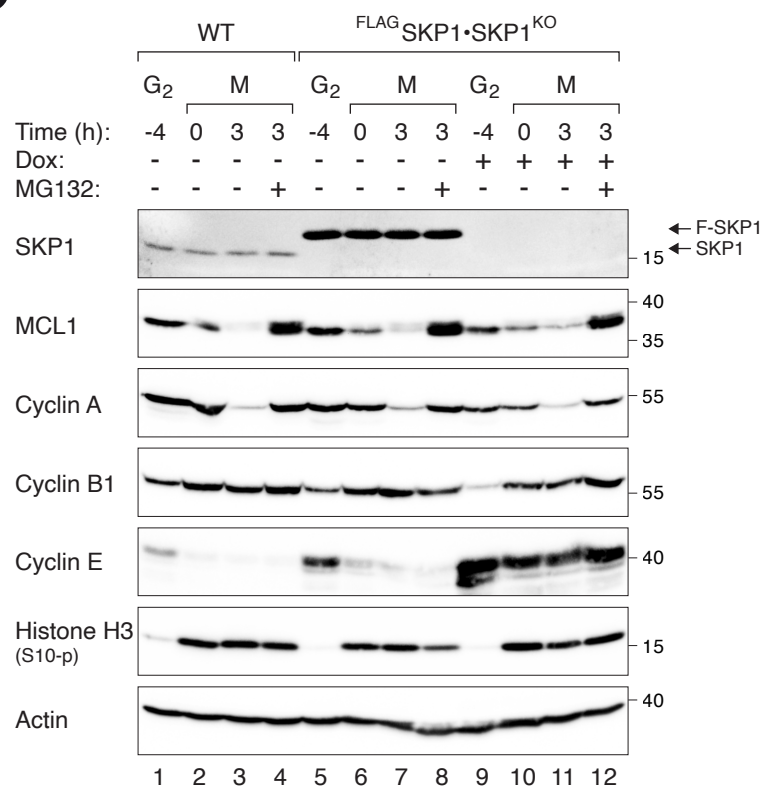

Supplement: Supplementary file 2 — Supplemental Figure S1 [file 41418_2022_1080_MOESM2_ESM.pdf]

A

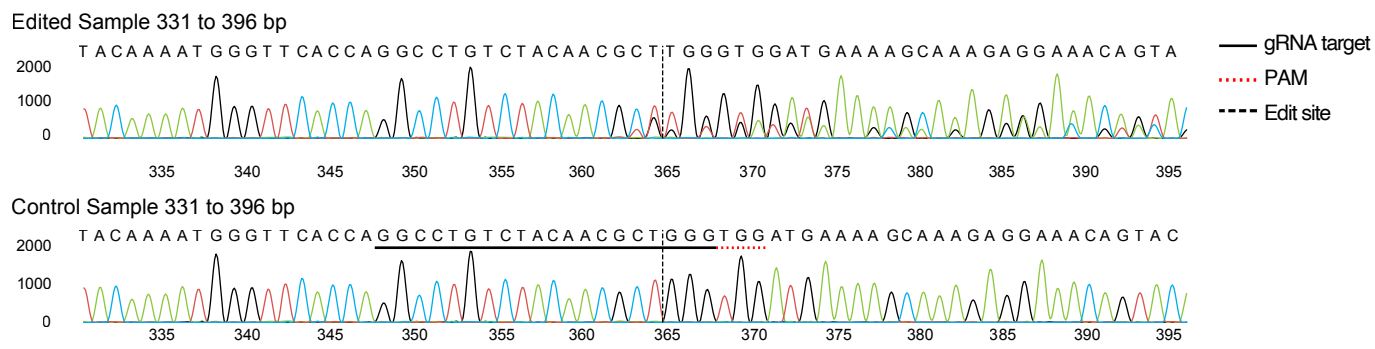

B

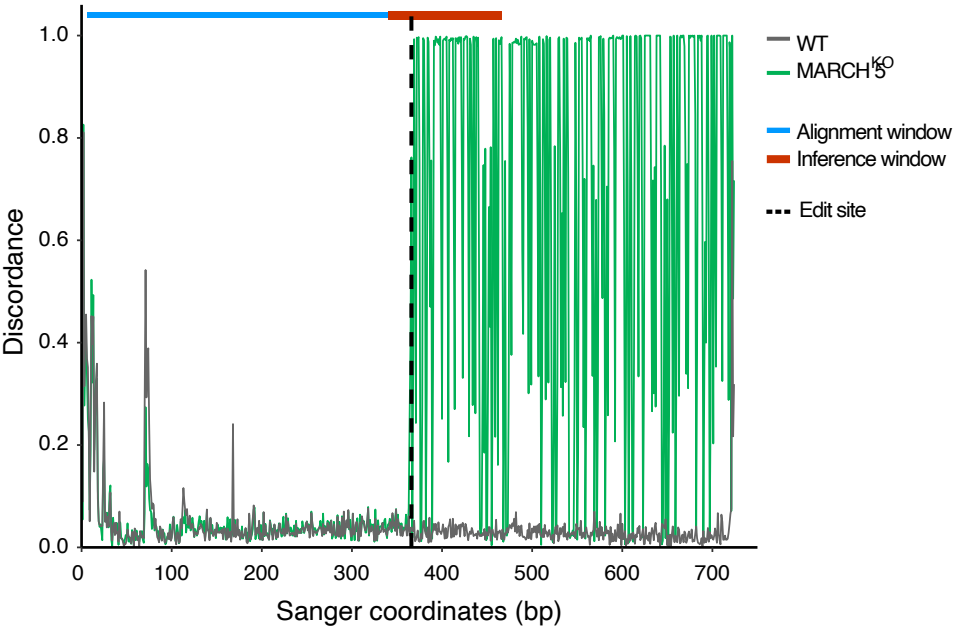

C

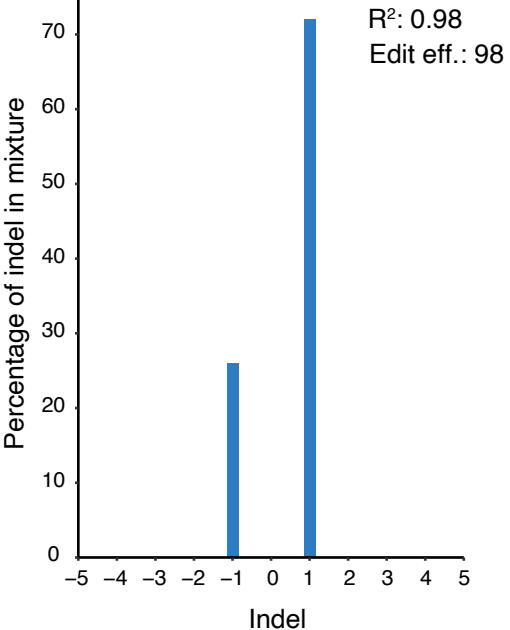

Supplemental Figure S2

Supplement: Supplementary file 3 — Supplemental Figure S2 [file 41418_2022_1080_MOESM3_ESM.pdf]

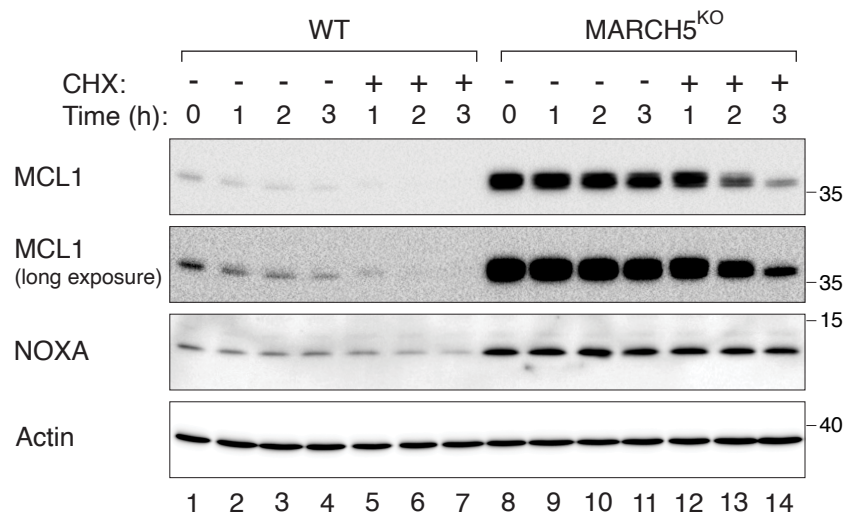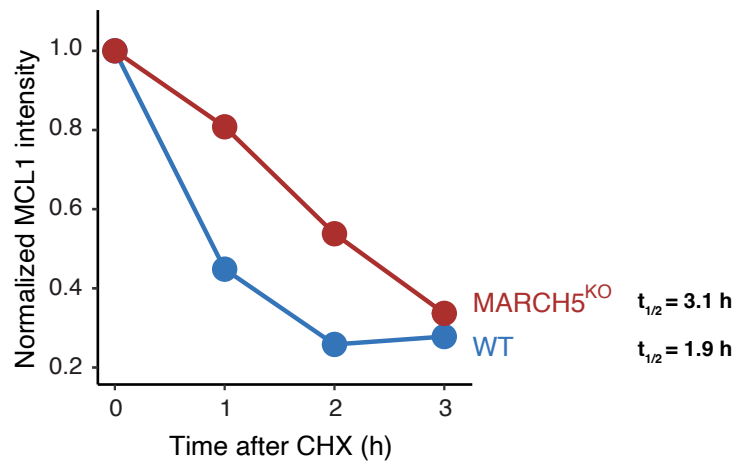

Supplemental Figure S3

Supplement: Supplementary file 4 — Supplemental Figure S3 [file 41418_2022_1080_MOESM4_ESM.pdf]

**A**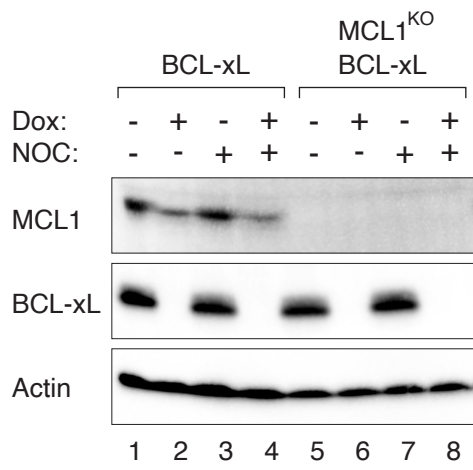**B**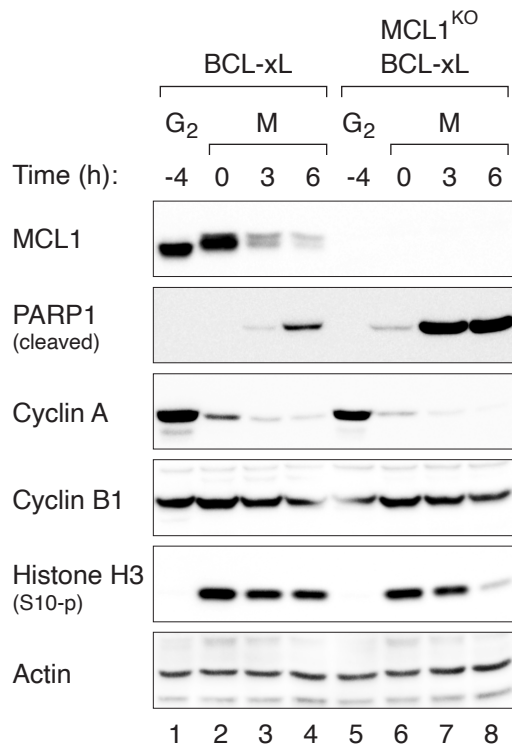**C**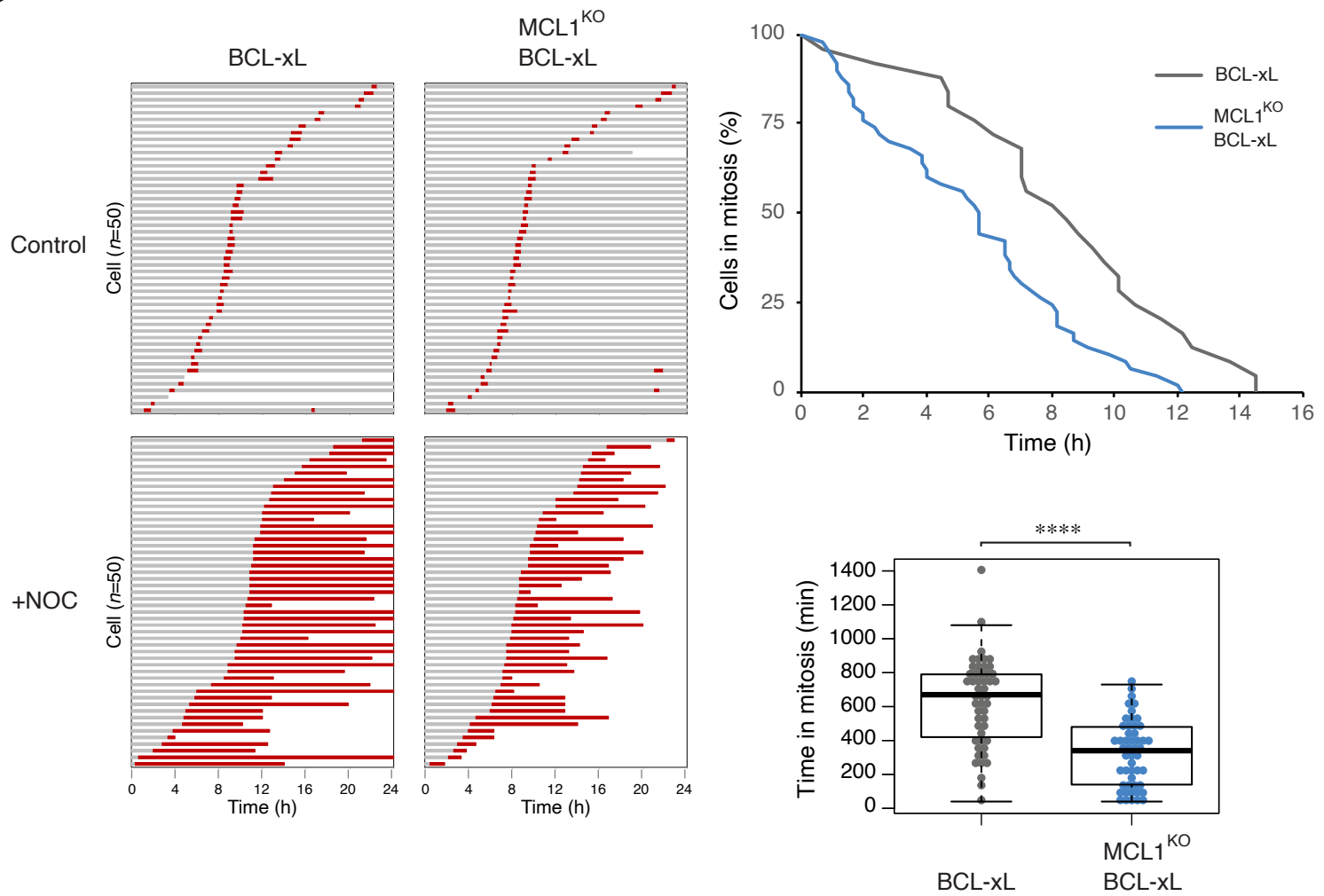

Supplemental Figure S4

Supplement: Supplementary file 5 — Supplemental Figure S4 [file 41418_2022_1080_MOESM5_ESM.pdf]

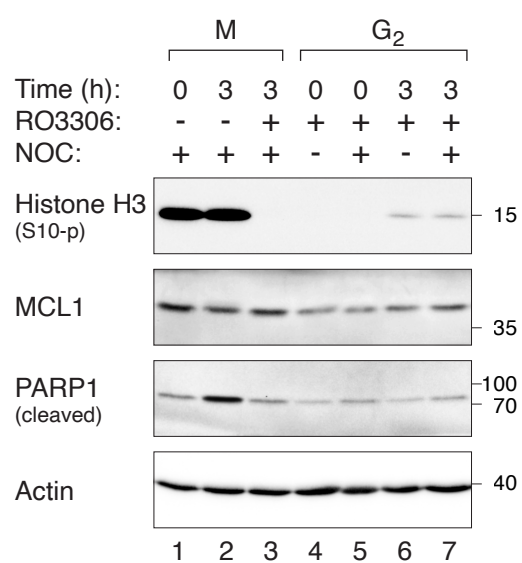

Supplemental Figure S5

Supplement: Supplementary file 6 — Supplemental Figure S5 [file 41418_2022_1080_MOESM6_ESM.pdf]

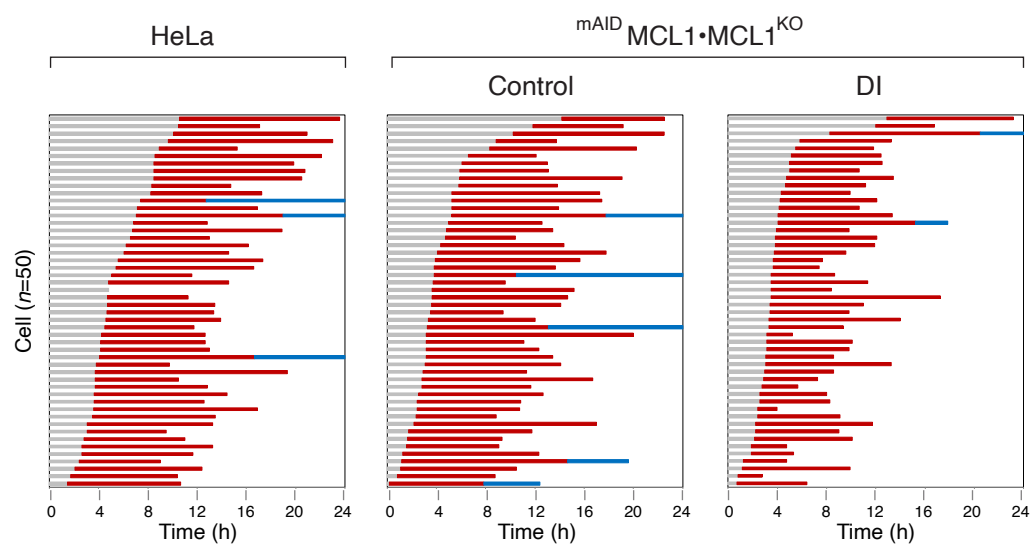

Supplemental Figure S6

Supplement: Supplementary file 7 — Supplemental Figure S6 [file 41418_2022_1080_MOESM7_ESM.pdf]

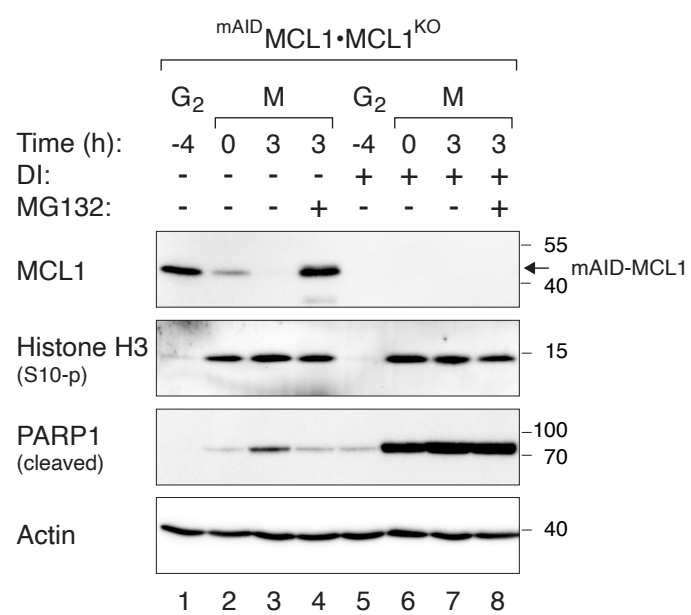

Supplemental Figure S7

Supplement: Supplementary file 8 — Supplemental Figure S7 [file 41418_2022_1080_MOESM8_ESM.pdf]

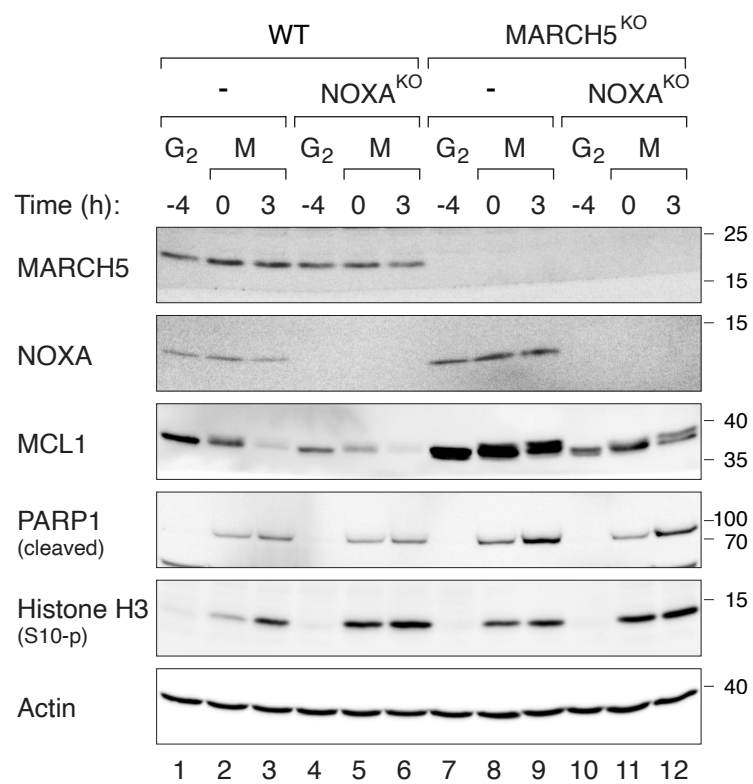

Supplemental Figure S8

Supplement: Supplementary file 9 — Supplemental Figure S8 [file 41418_2022_1080_MOESM9_ESM.pdf]

**A**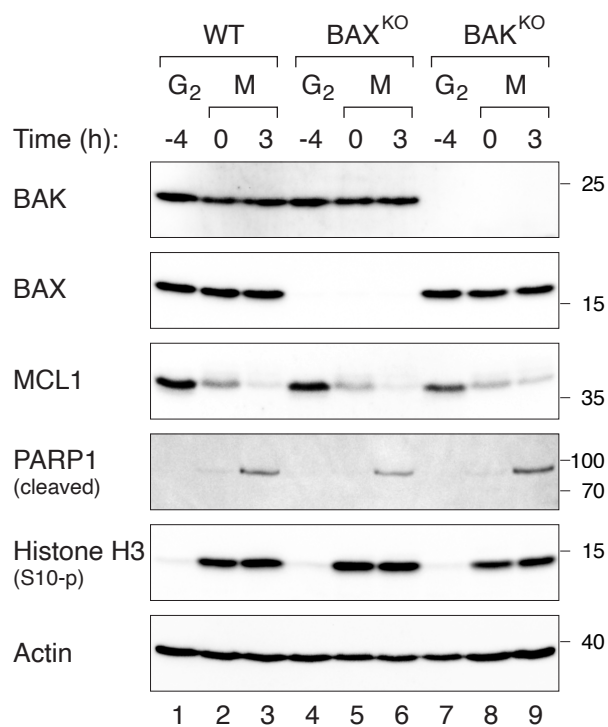**B**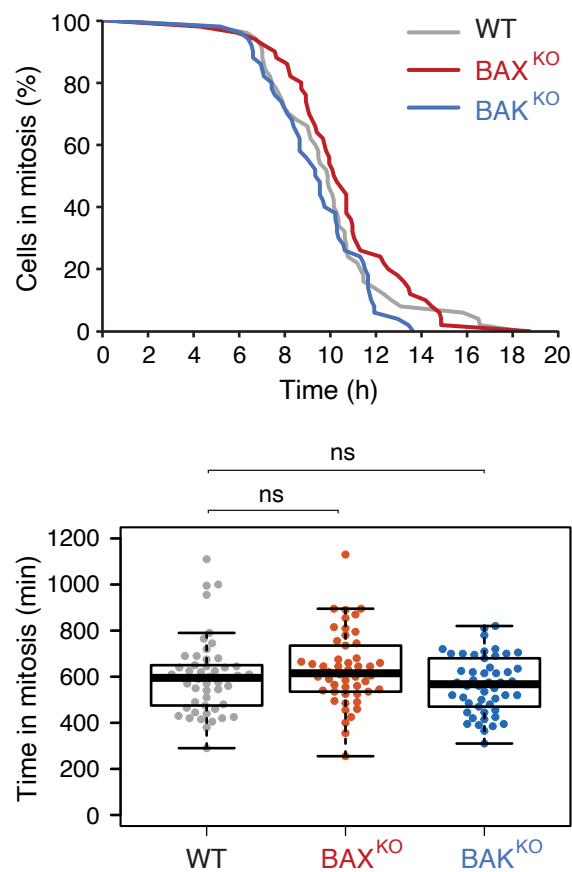**C**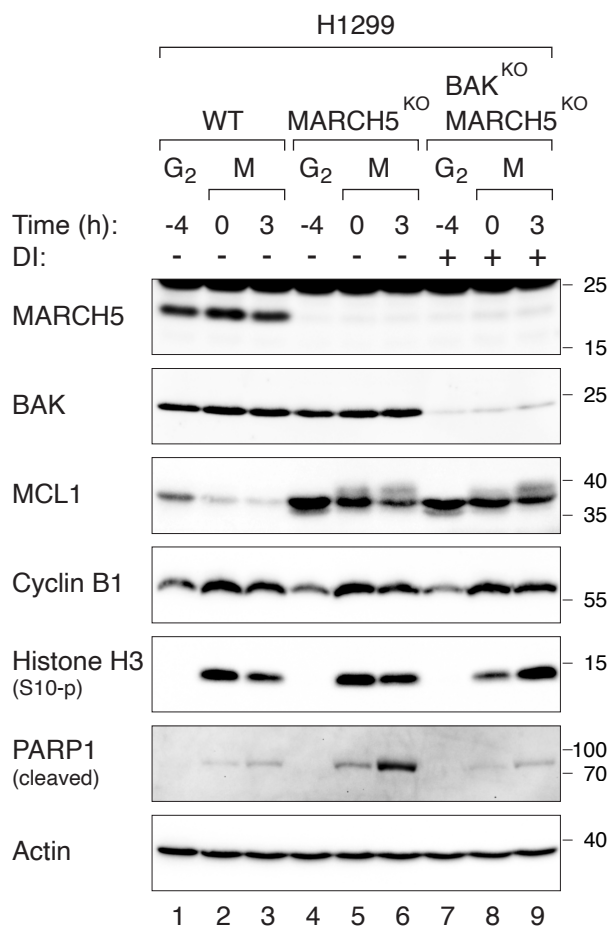

Supplement: Supplementary file 10 — Supplemental Figure S9 [file 41418_2022_1080_MOESM10_ESM.pdf]

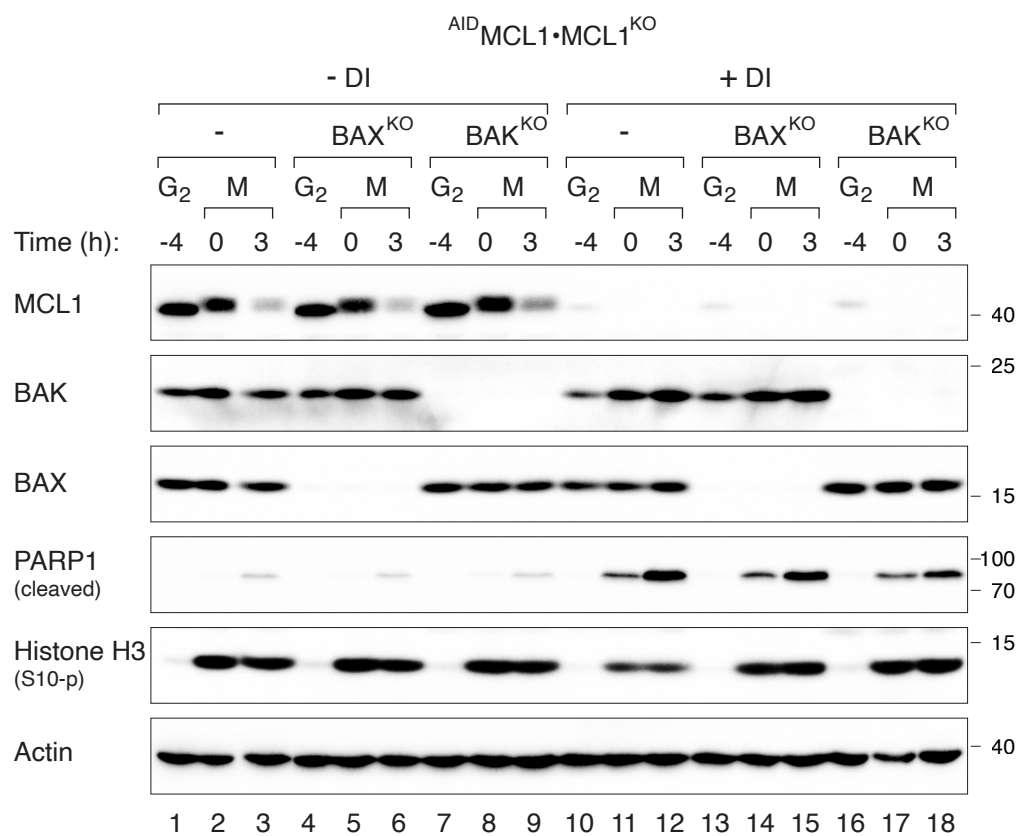

Supplemental Figure S10

Supplement: Supplementary file 11 — Supplemental Figure S10 [file 41418_2022_1080_MOESM11_ESM.pdf]

# A

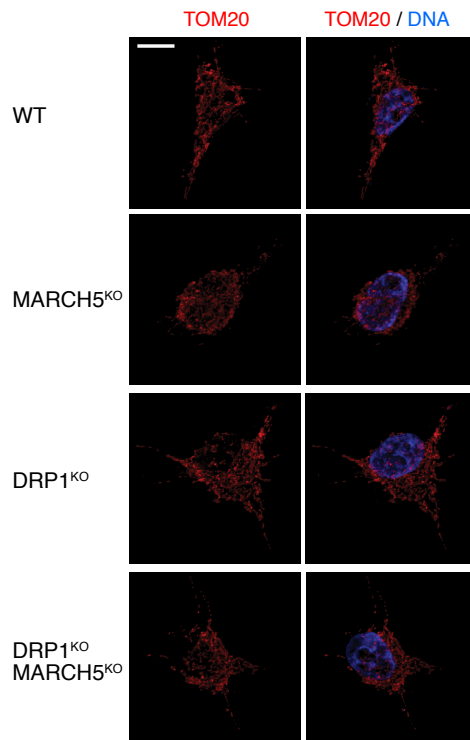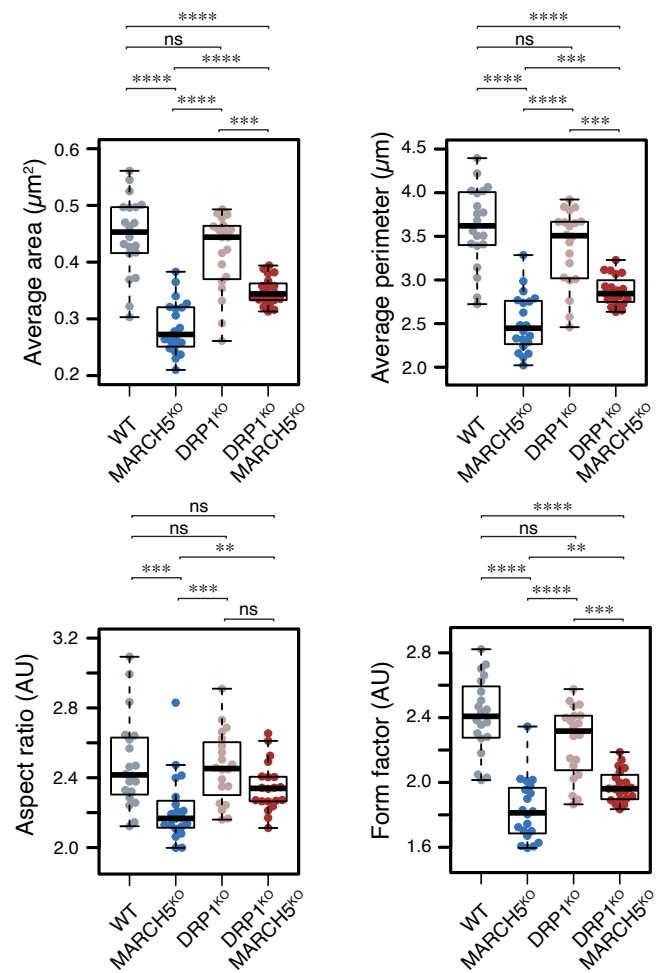

# B

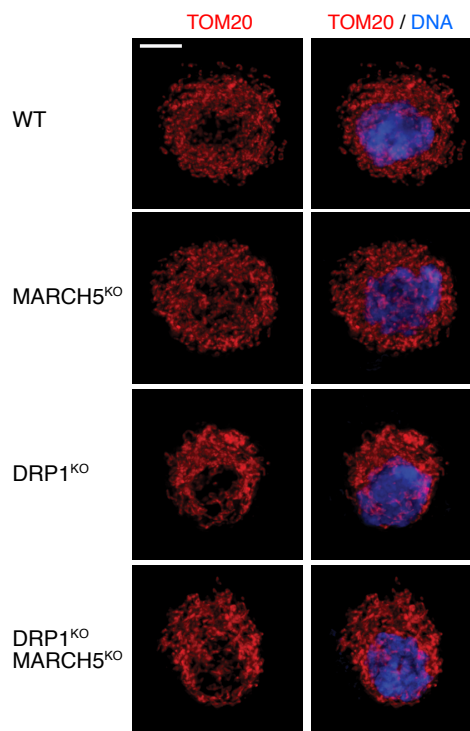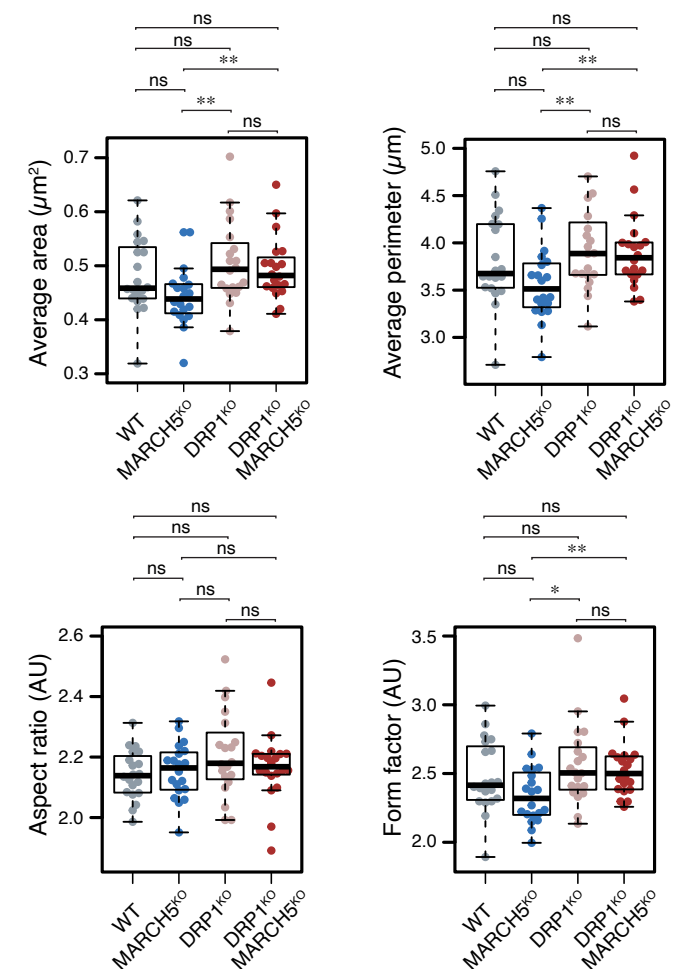

Supplemental Figure S11

Supplement: Supplementary file 12 — Supplemental Figure S11 [file 41418_2022_1080_MOESM12_ESM.pdf]
