# Supplementary material for: MARCH5 regulates mitotic apoptosis through MCL1-dependent and independent mechanisms
Source: Cell Death Differ. 2022 Nov 3;30(3):753–65. doi: 10.1038/s41418-022-01080-2 (PMC9984497; doi:10.1038/s41418-022-01080-2)
Supplement: Supplementary file 13 — Supplemental Figure S12 [file 41418_2022_1080_MOESM13_ESM.pdf]

Supplemental materials: original Western blots

Boxes indicate the lanes and bands used in the Figures and Supplemental Figures.
All lanes and bands were used if no box is indicated.

Fig 1A

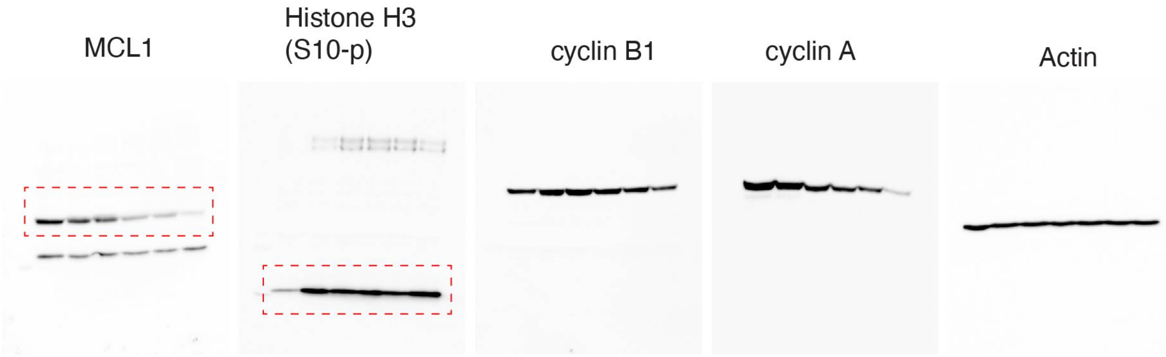

Fig 1C

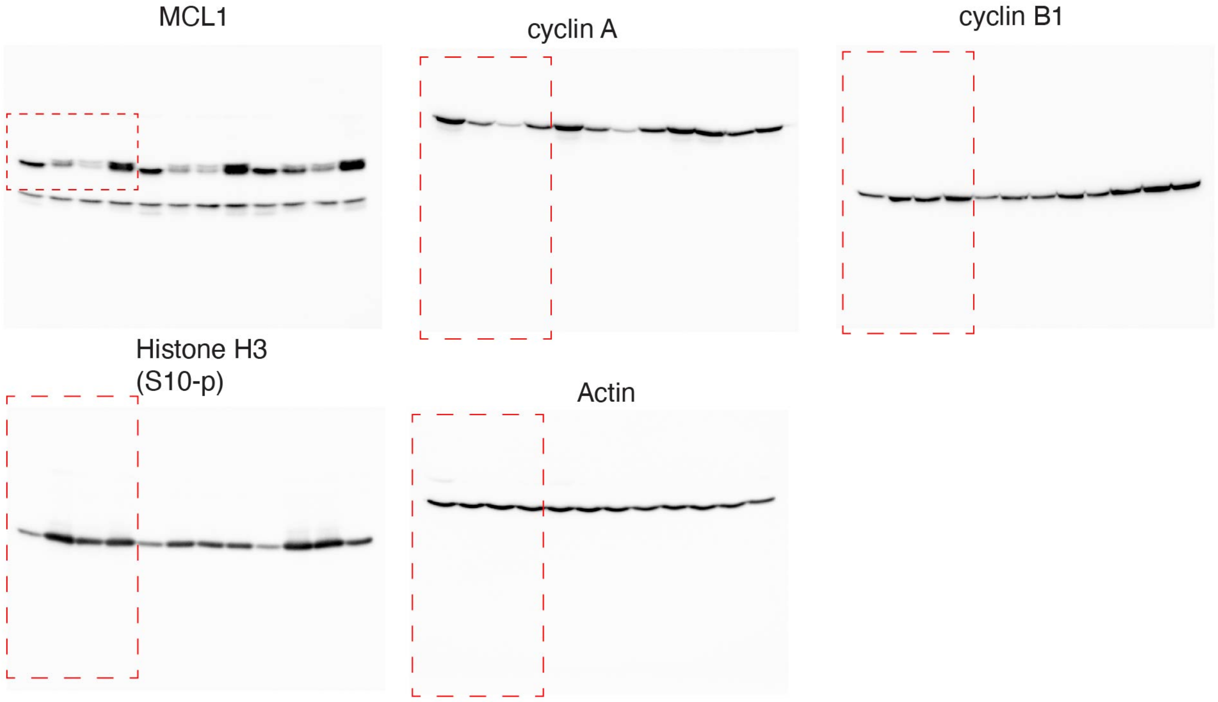

Fig 1D

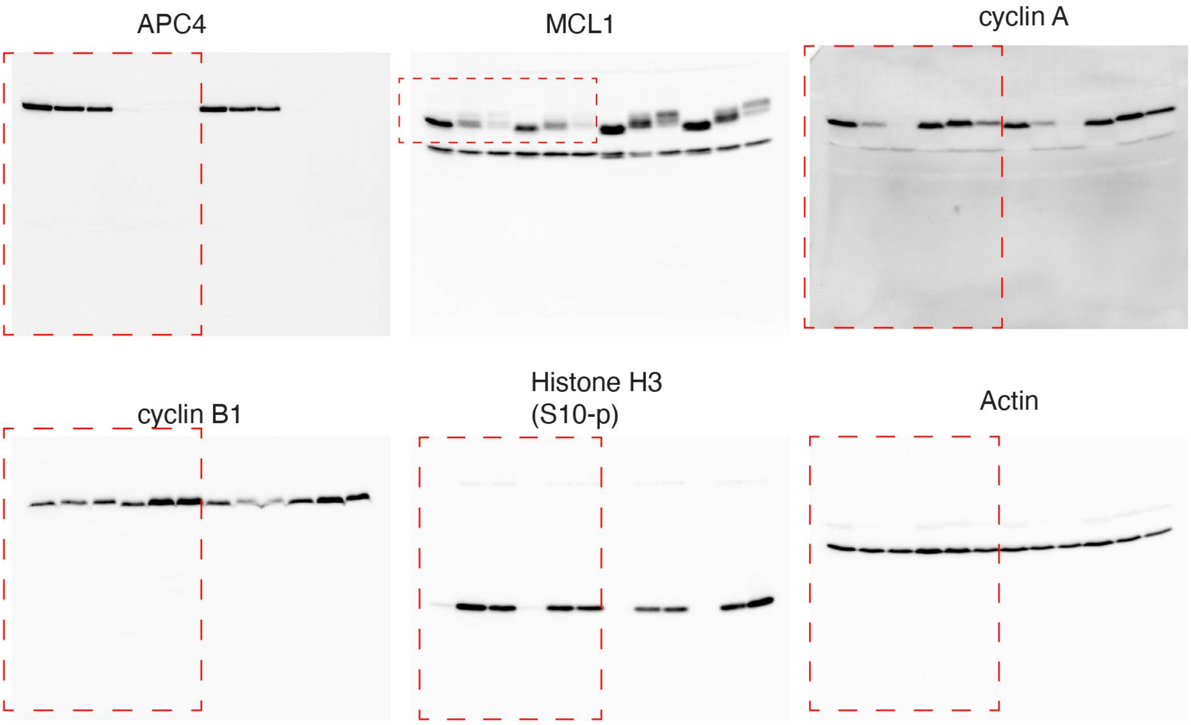

Original Western blots of Figure 1

Fig 2A

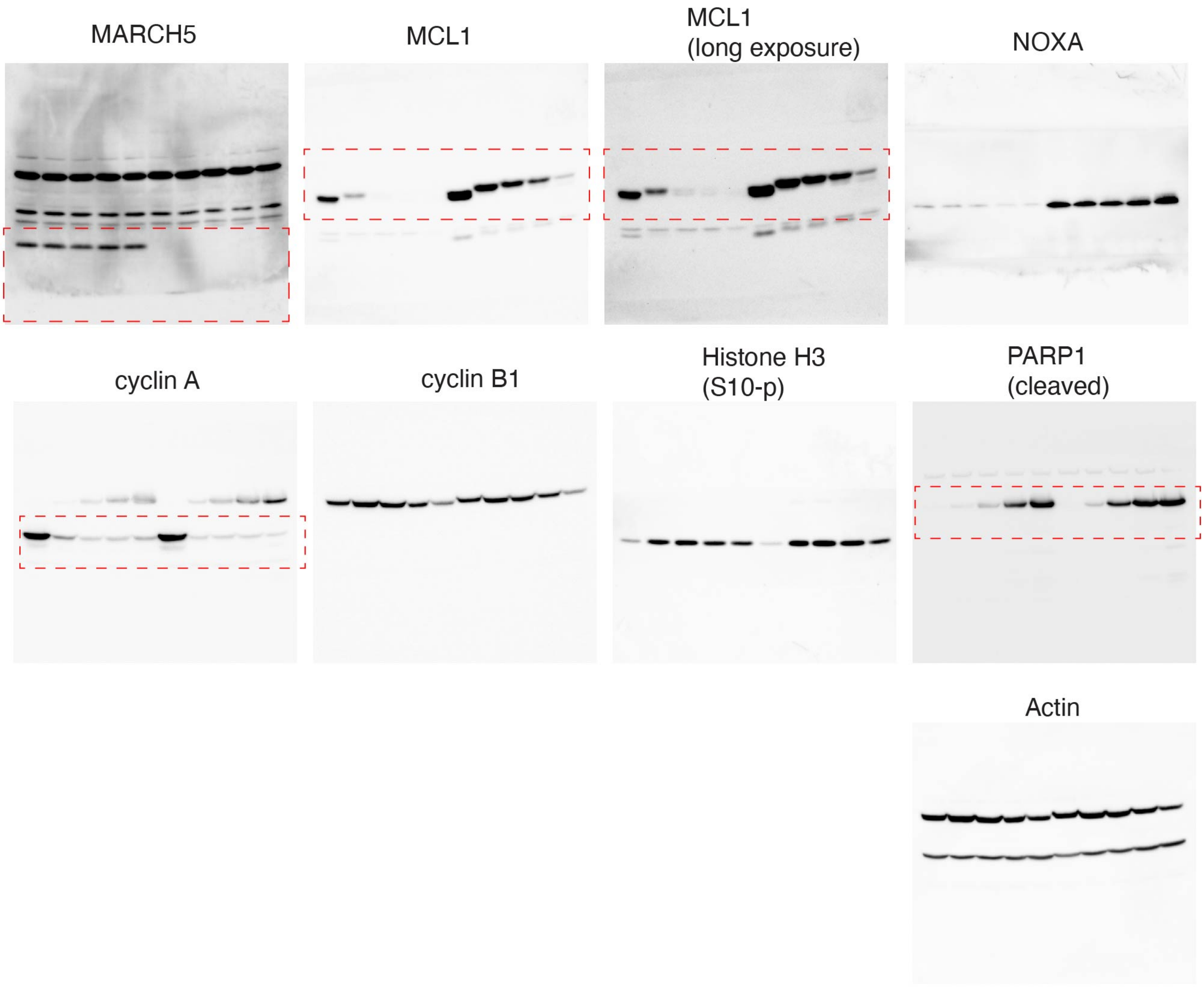

Fig 2B

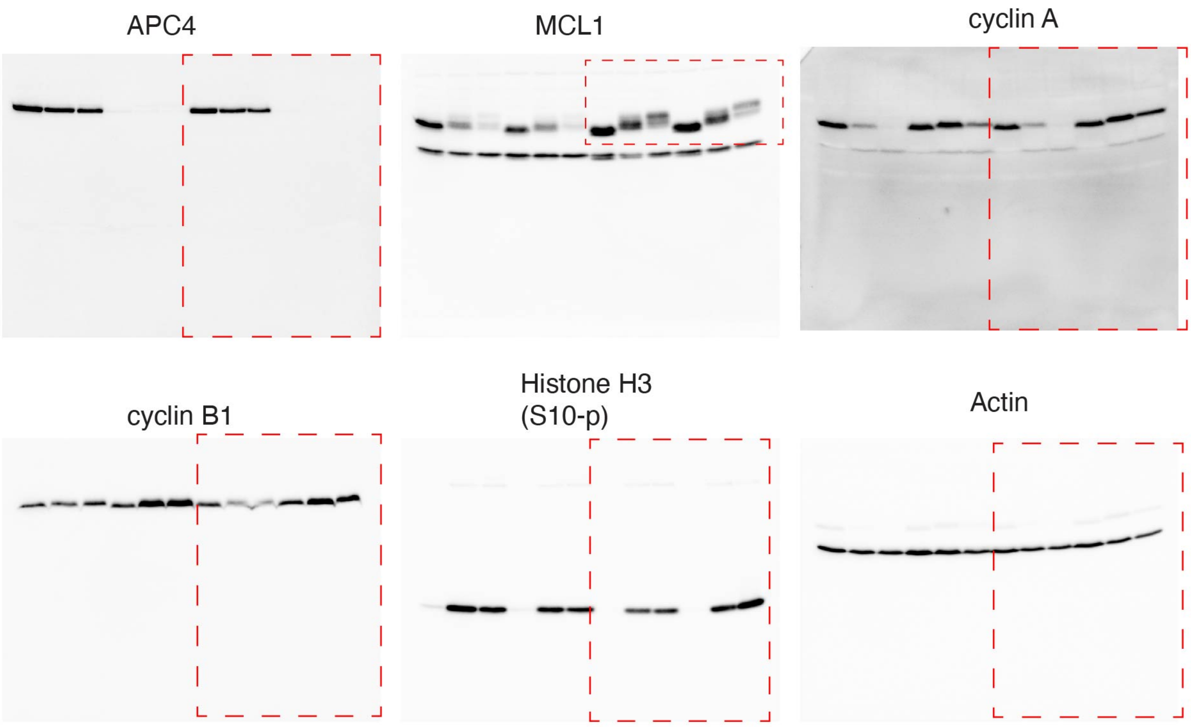

Original Western blots of Figure 2

Fig 2C

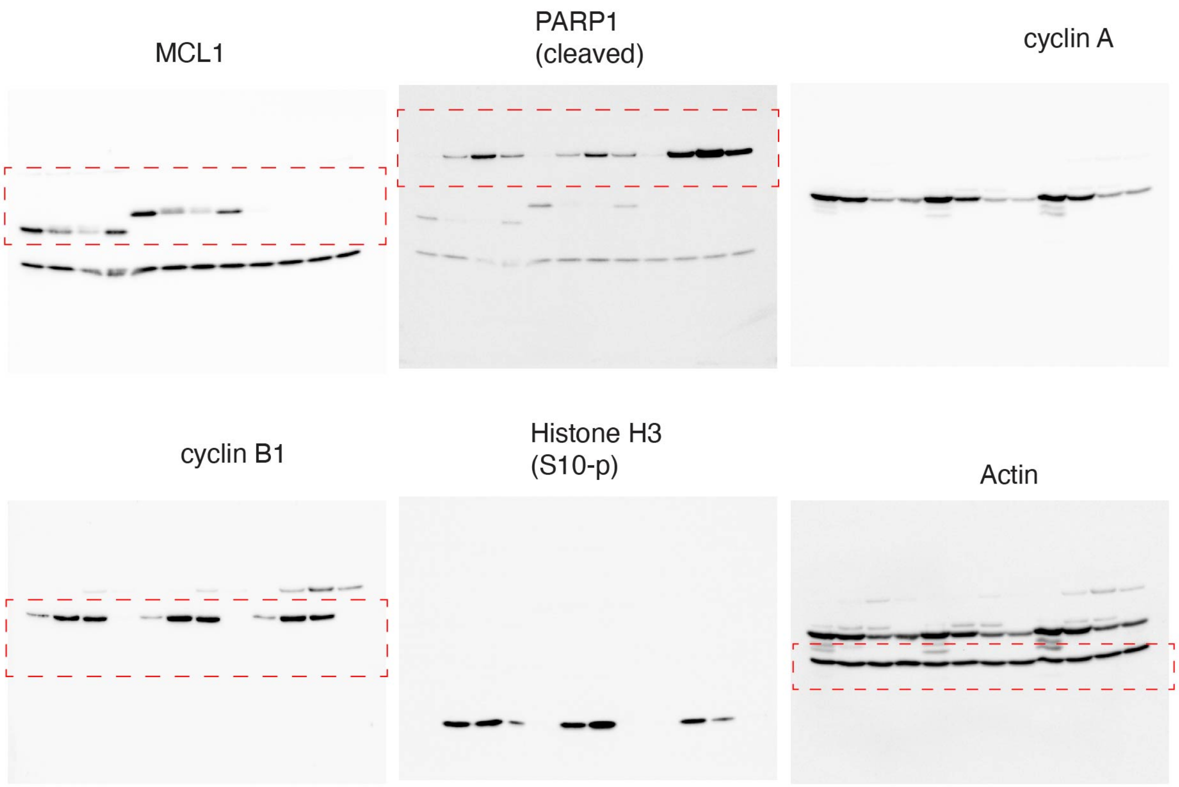

Fig 3B

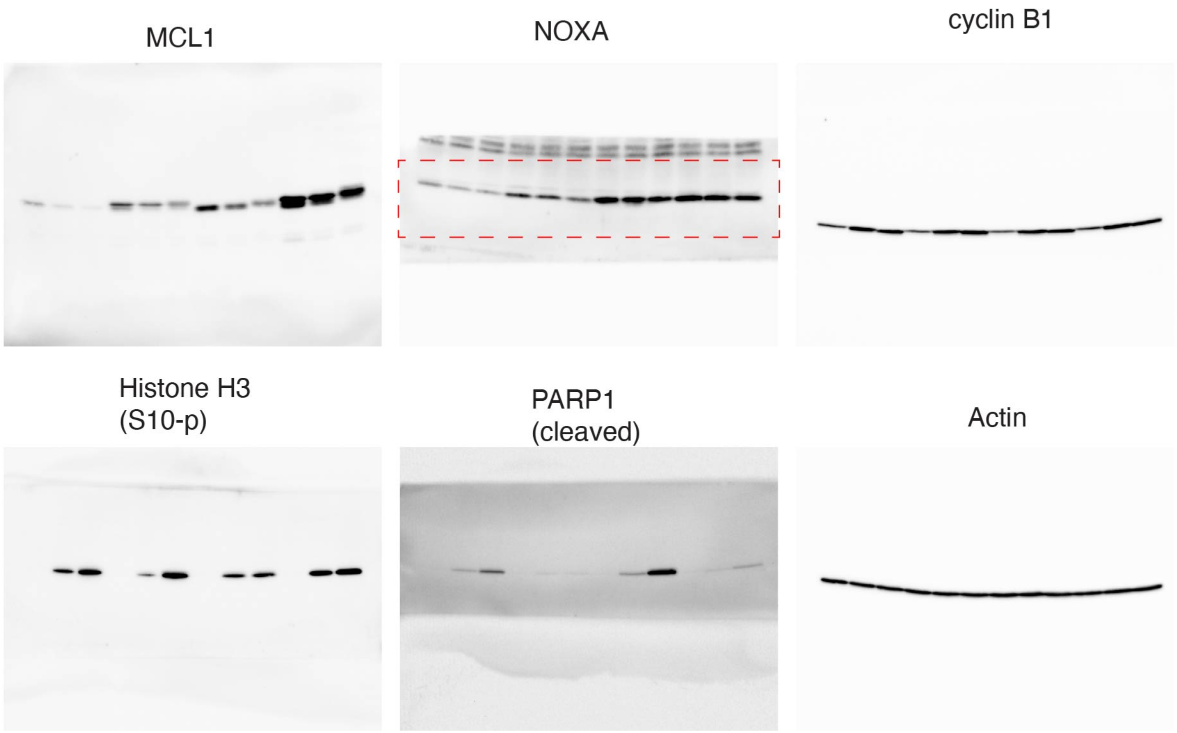

Fig 3C

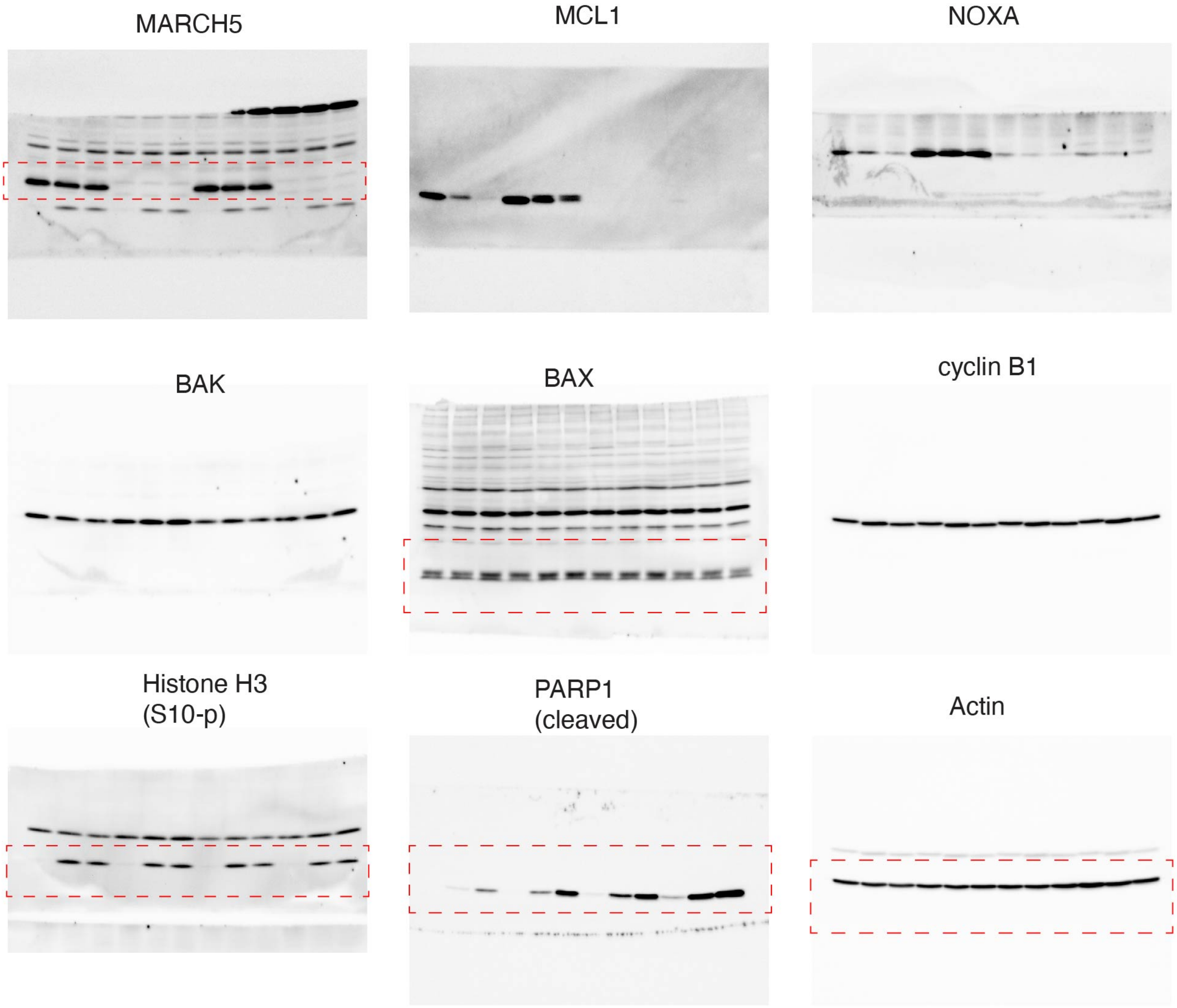

Original Western blots of Figure 3

Fig 4A

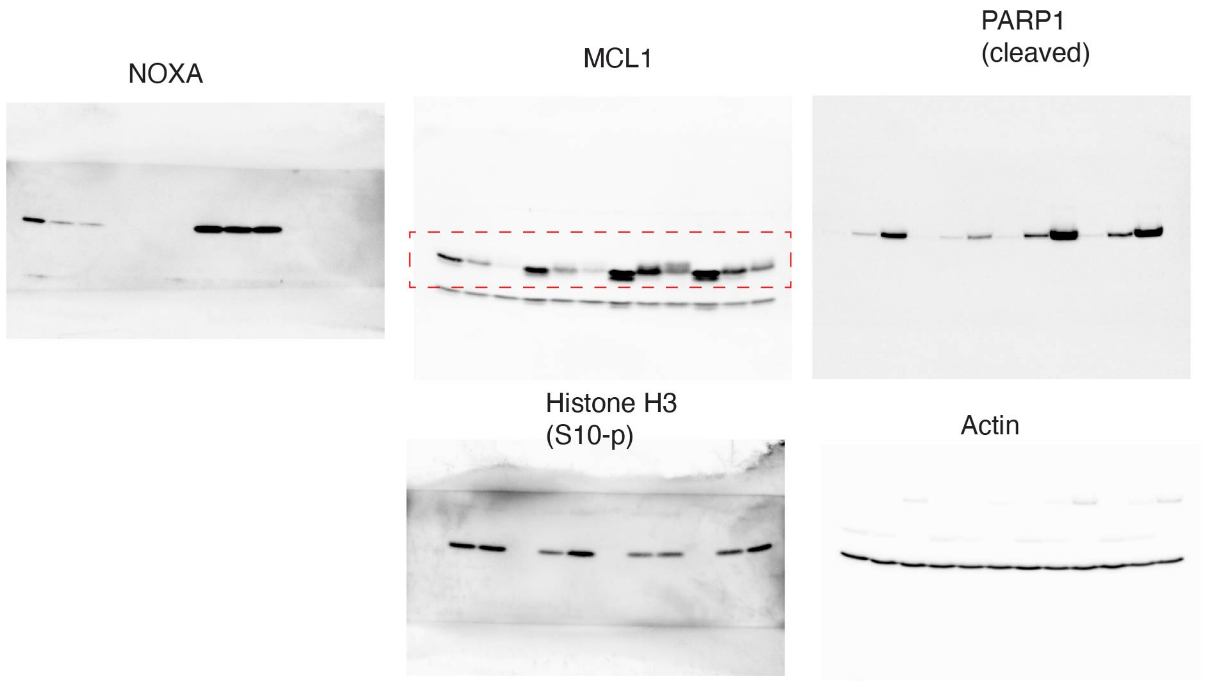

Fig 4C

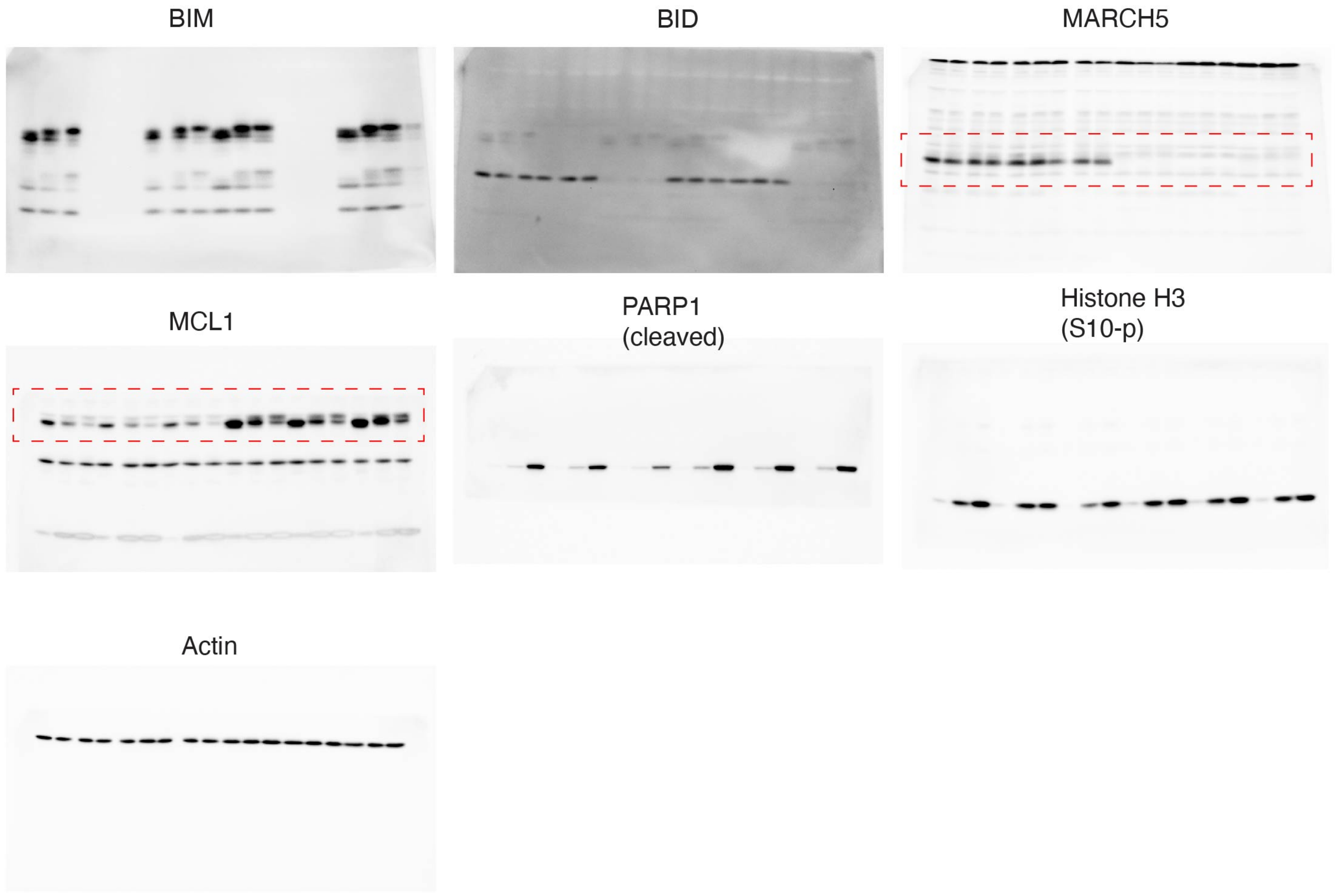

Original Western blots of Figure 4

Fig 5A

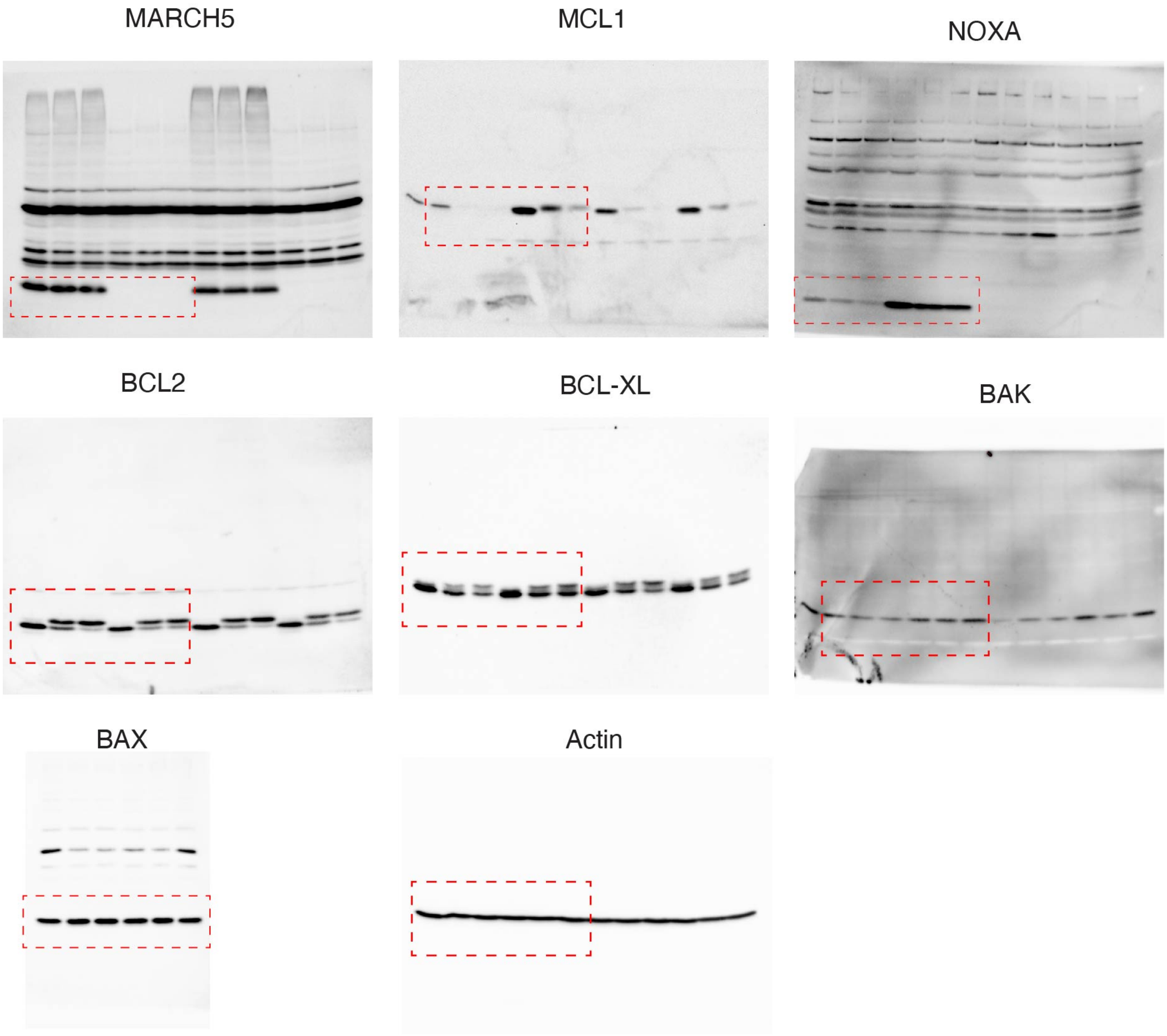

Fig 5C

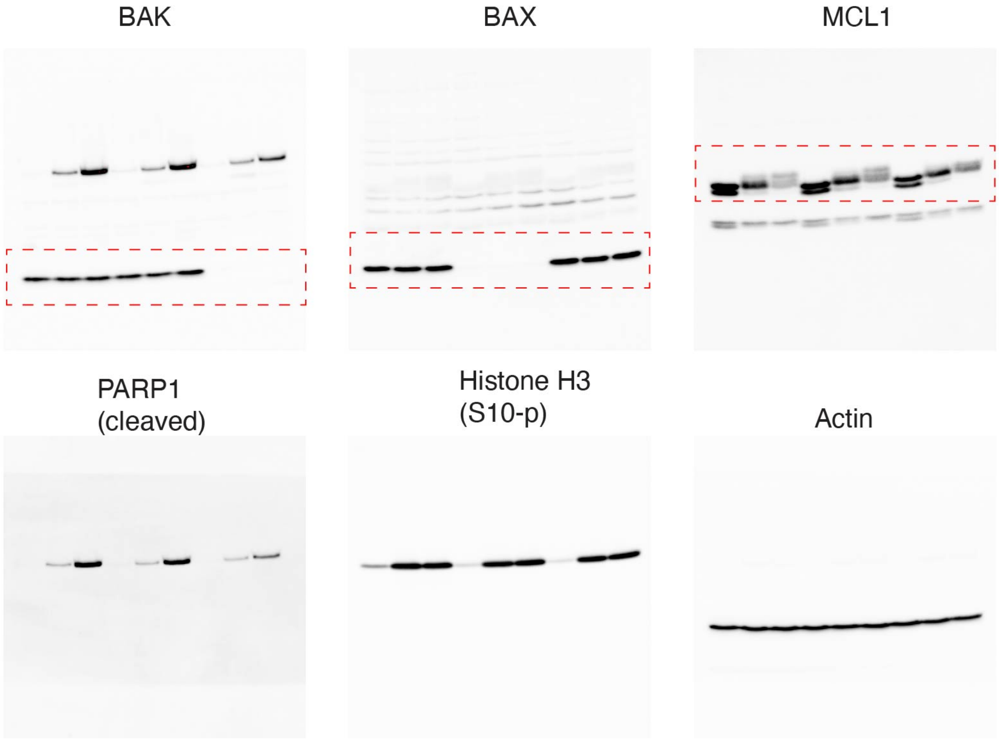

Original Western blots of Figure 5

Fig 6A

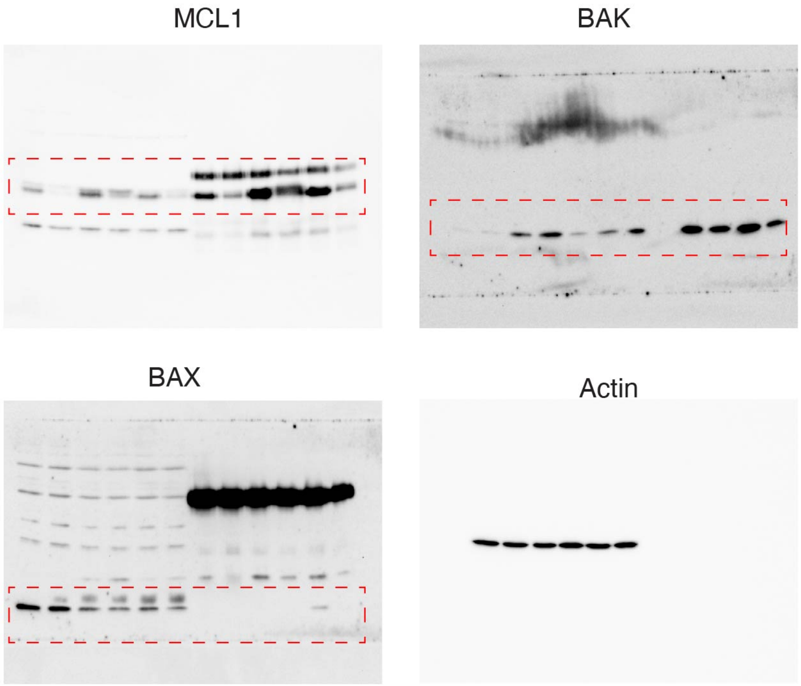

Fig 6B

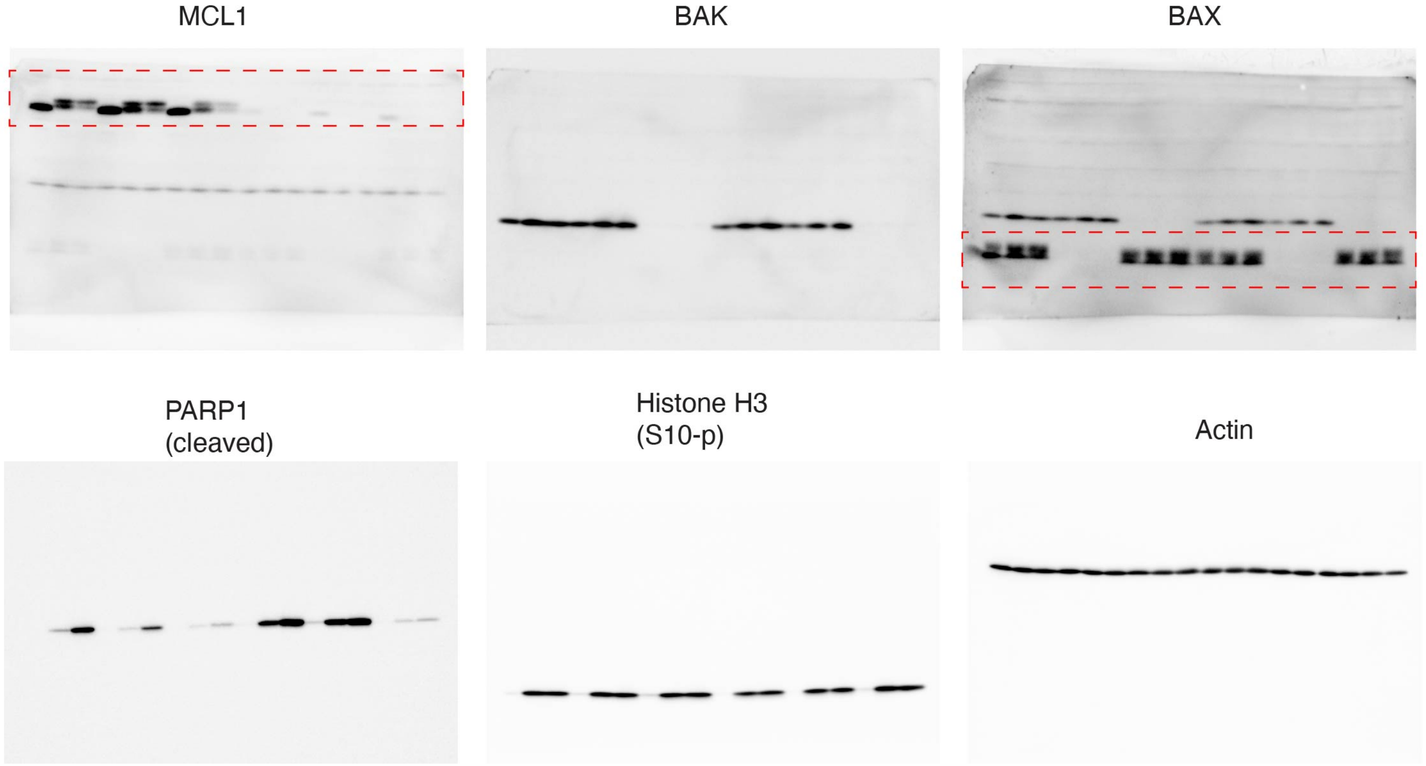

Original Western blots of Figure 6

Fig 7A

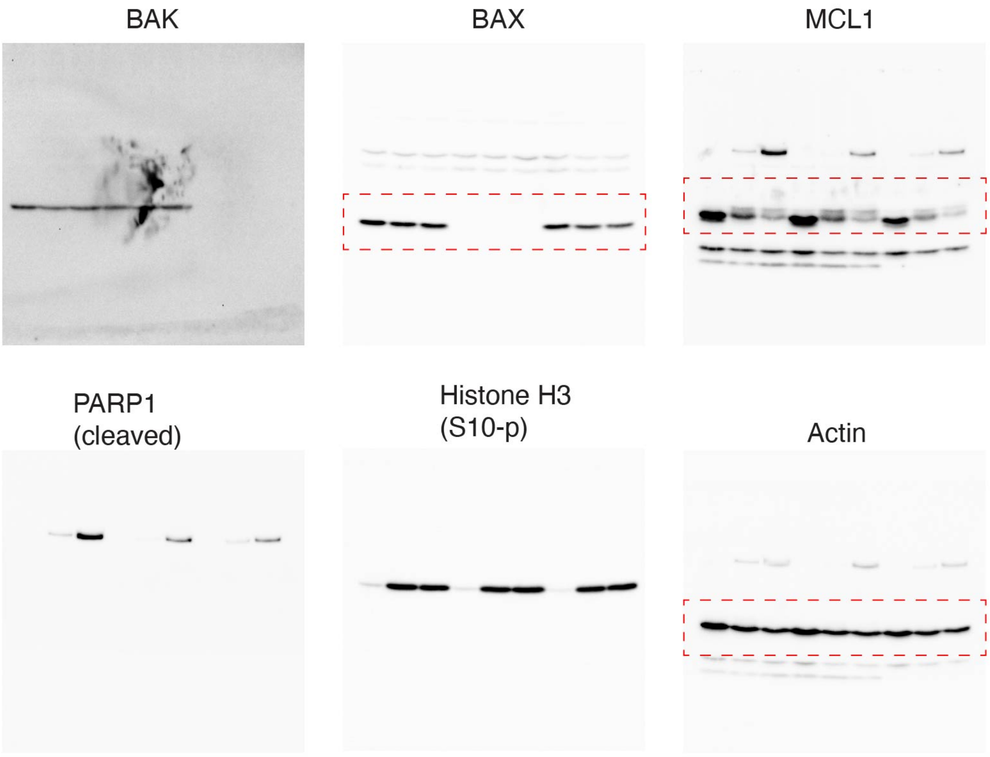

Fig 7B

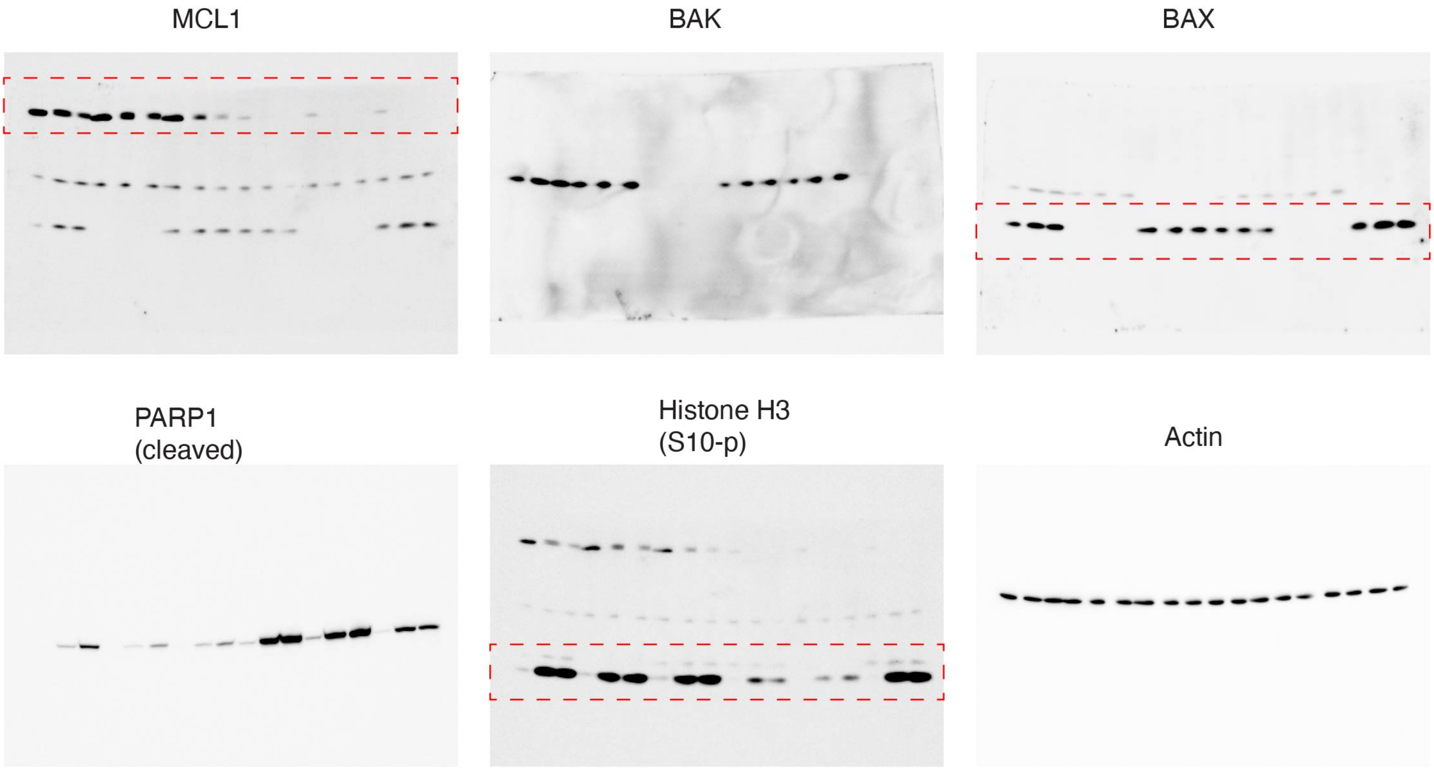

Original Western blots of Figure 7

Fig 8A

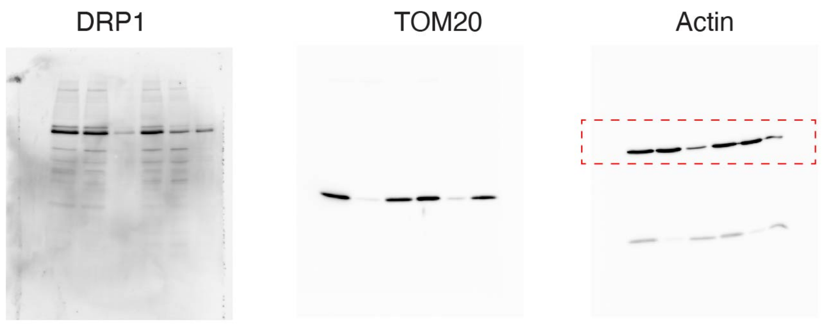

Fig 8B

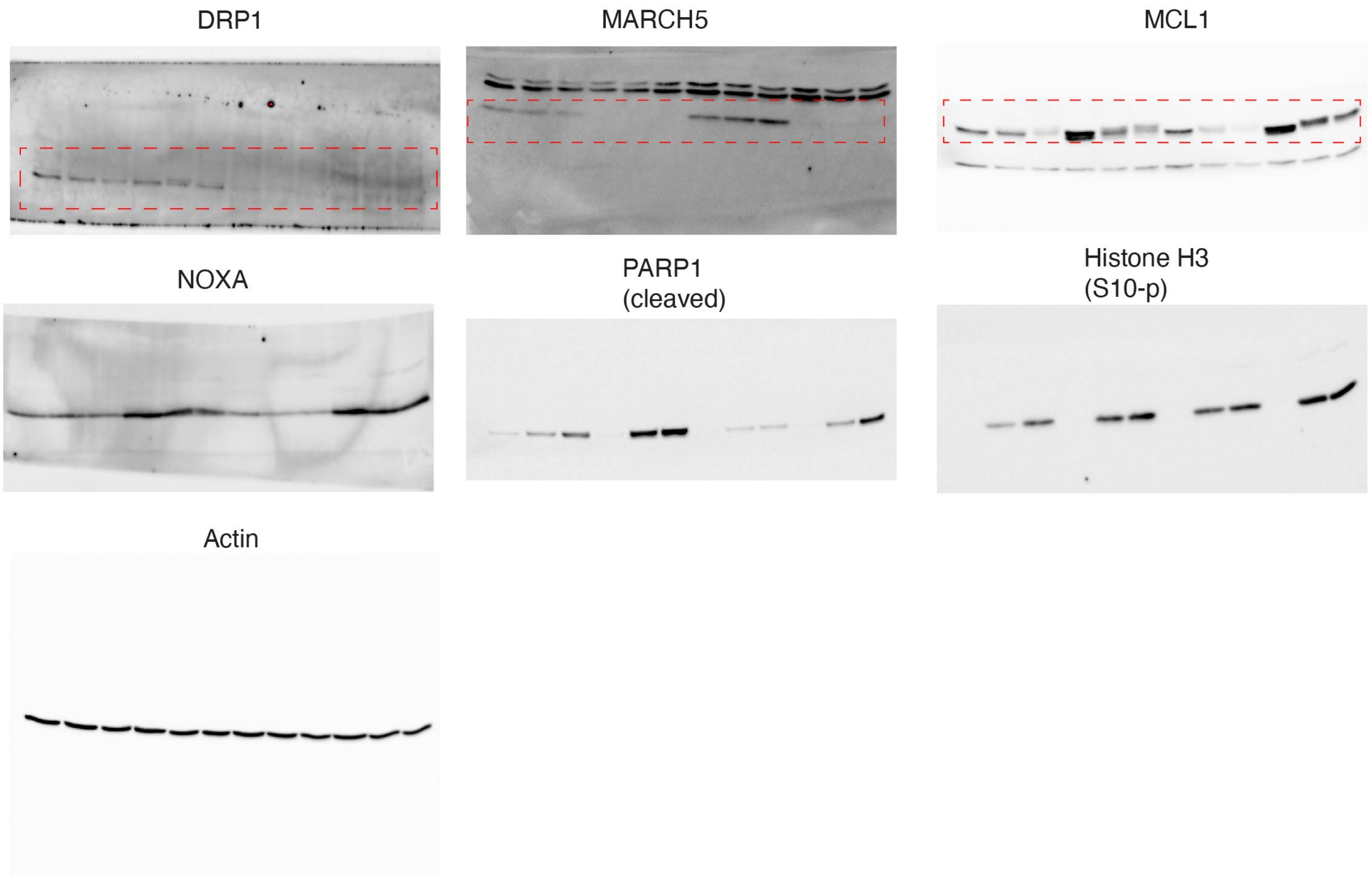

Fig 8C

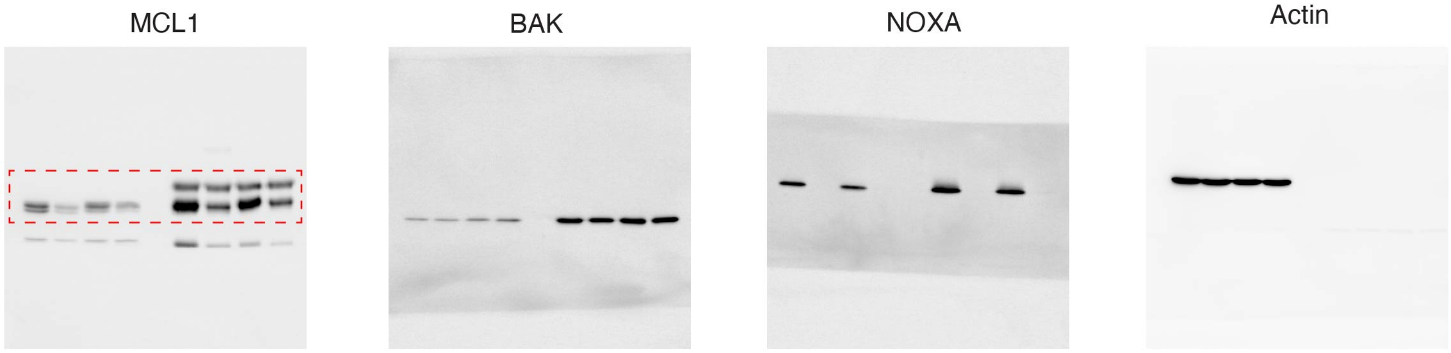

Original Western blots of Figure 8

Fig S1A

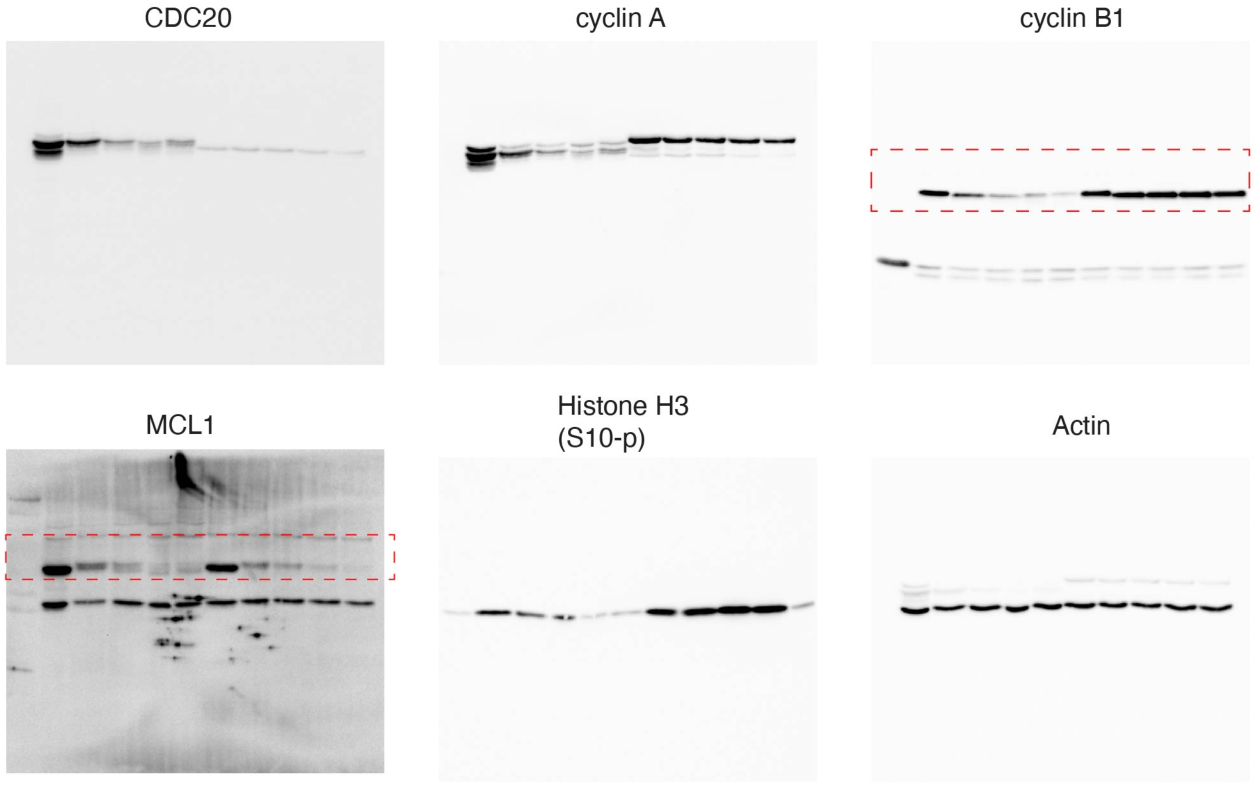

Fig S1B

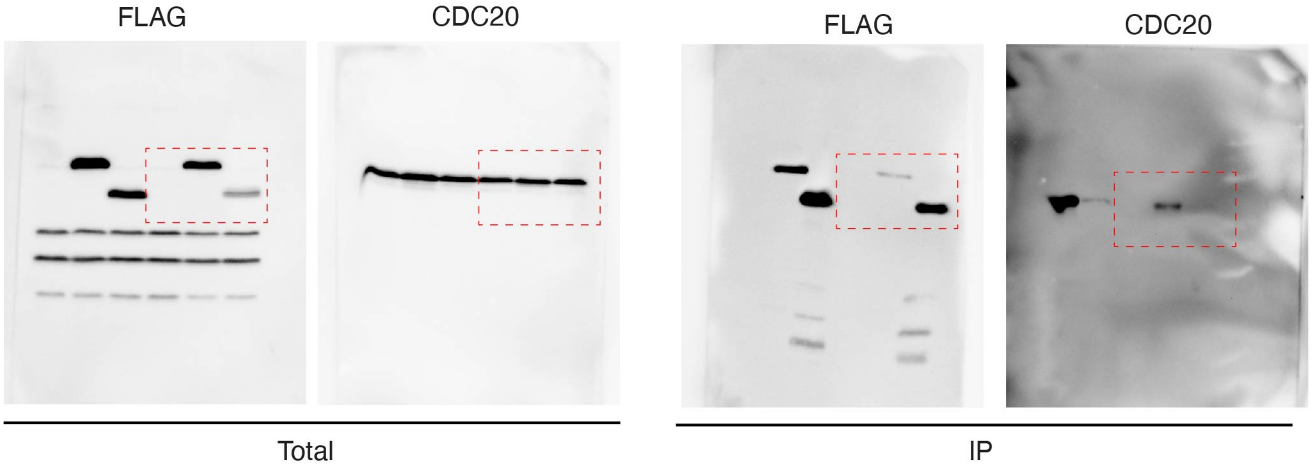

Fig S1C

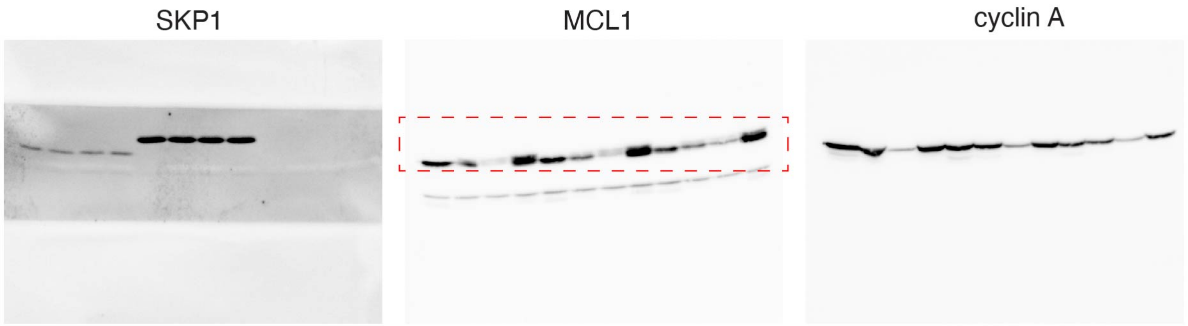

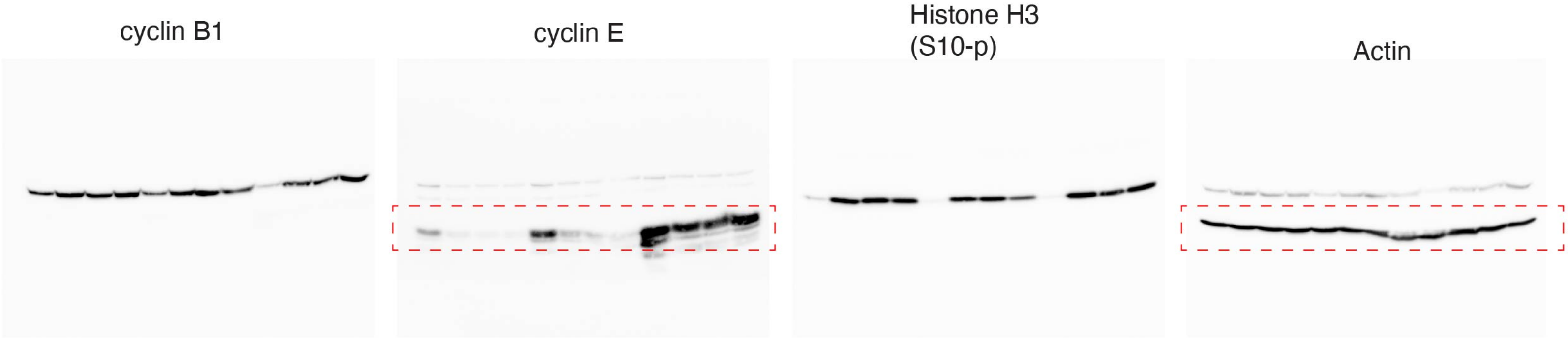

Original Western blots of Figure S1

Fig S3

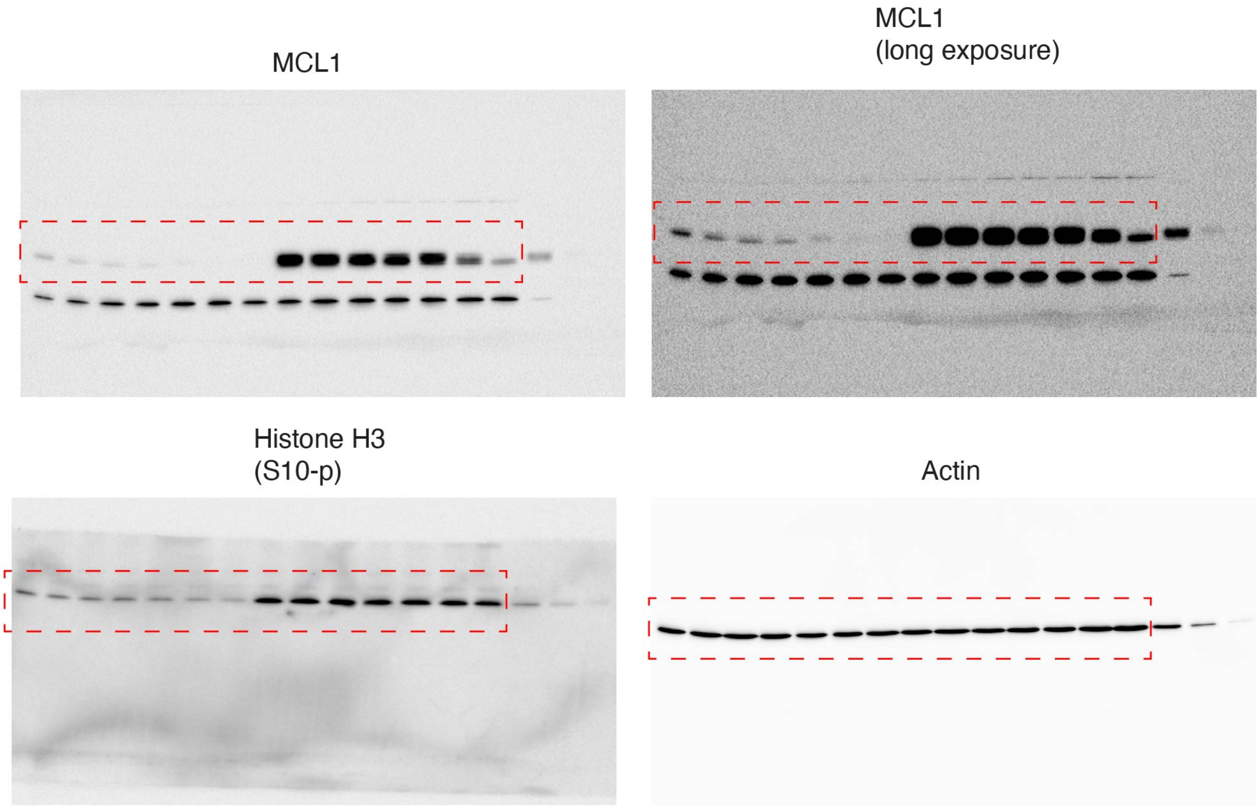

Original Western blots of Figure S3

Fig S4A

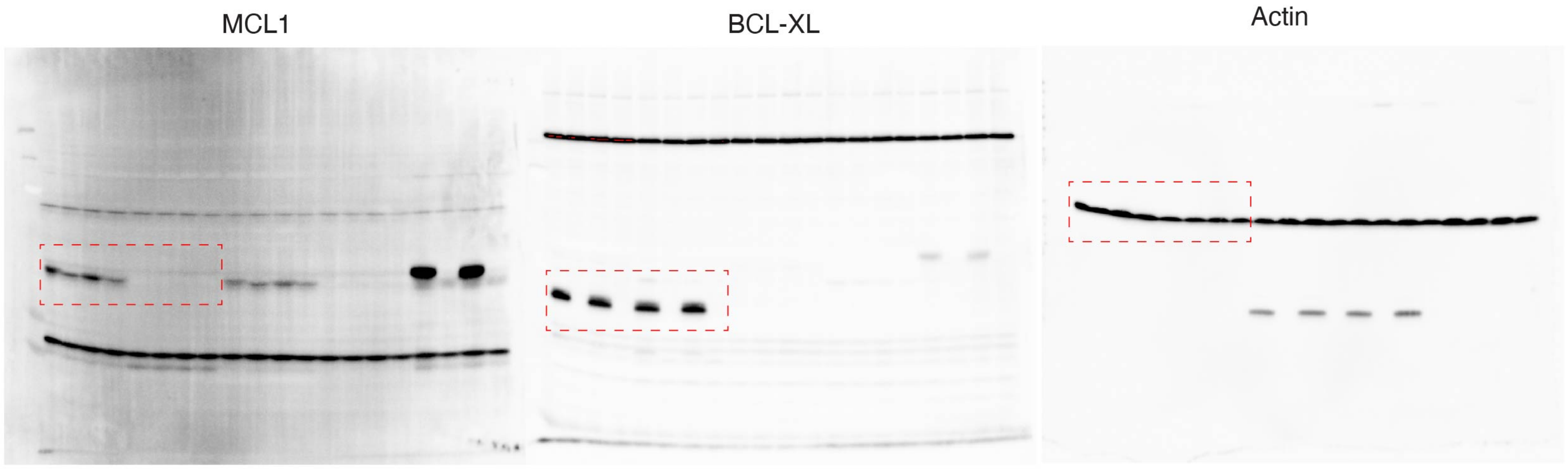

Fig S4B

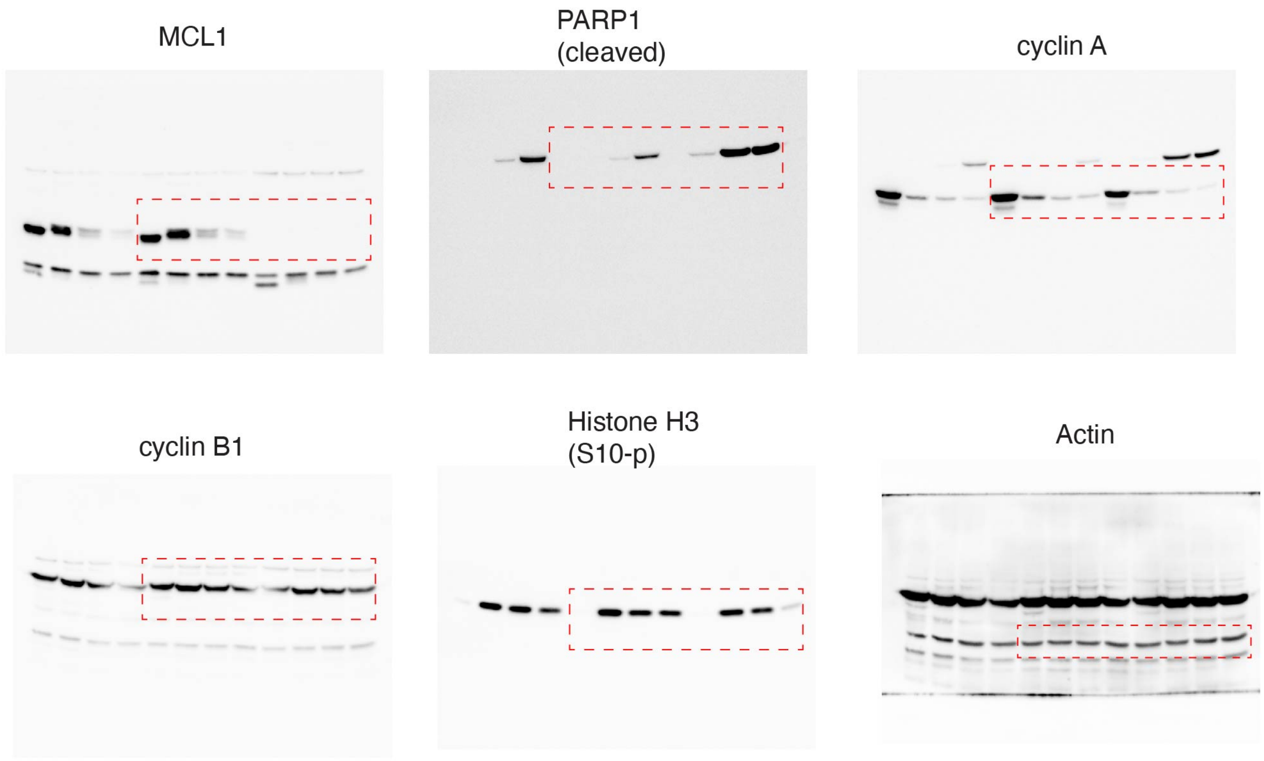

Original Western blots of Figure S4

Fig S5

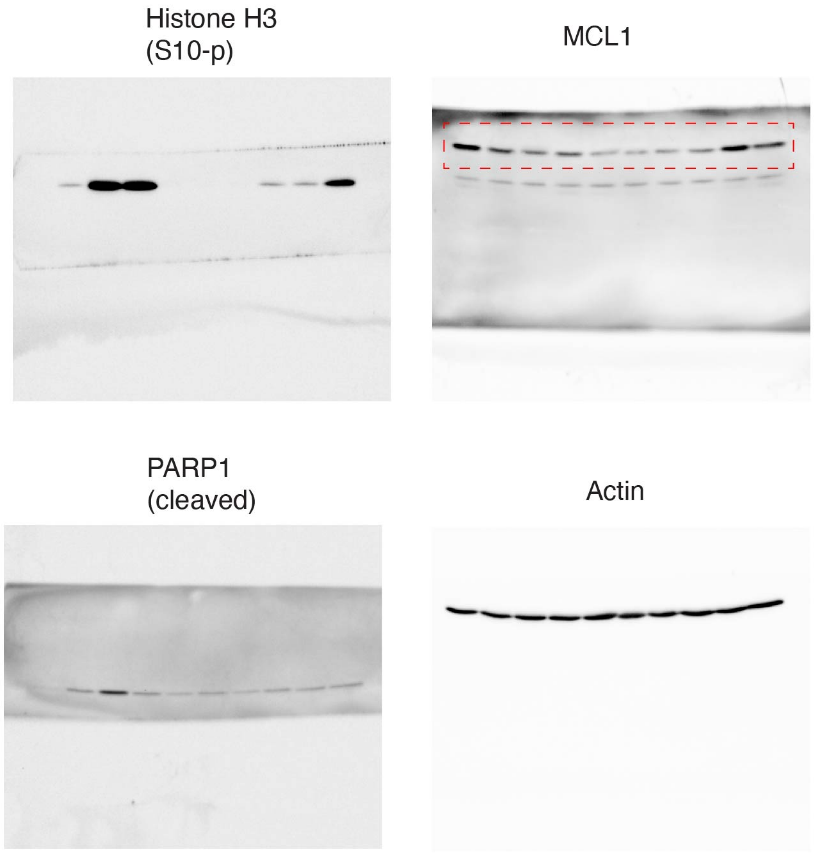

Original Western blots of Figure S5

Fig S7

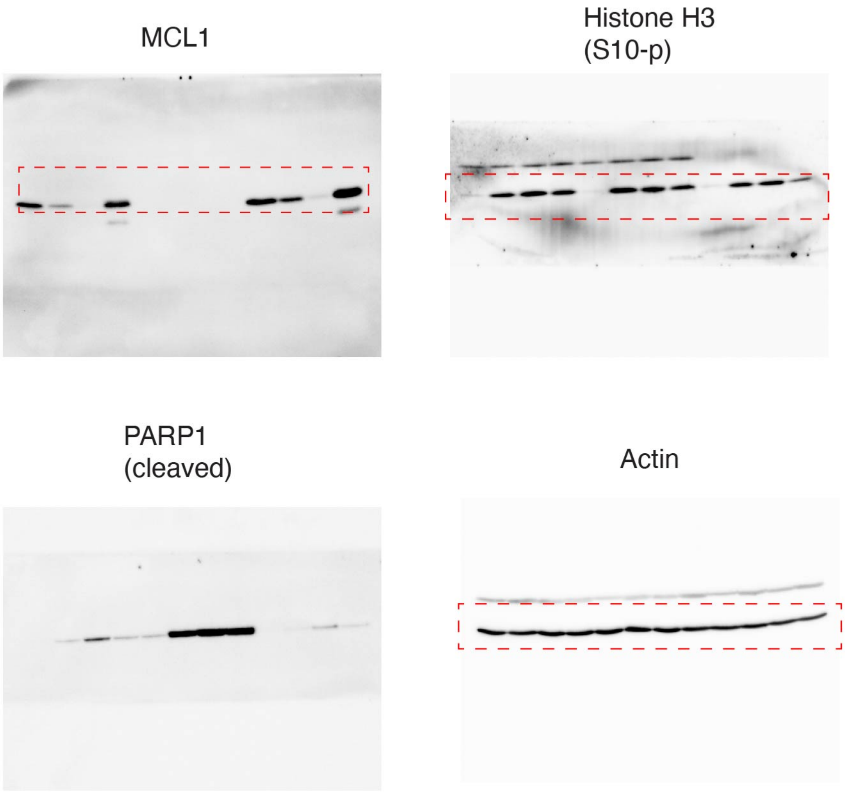

Original Western blots of Figure S7

Fig S9A

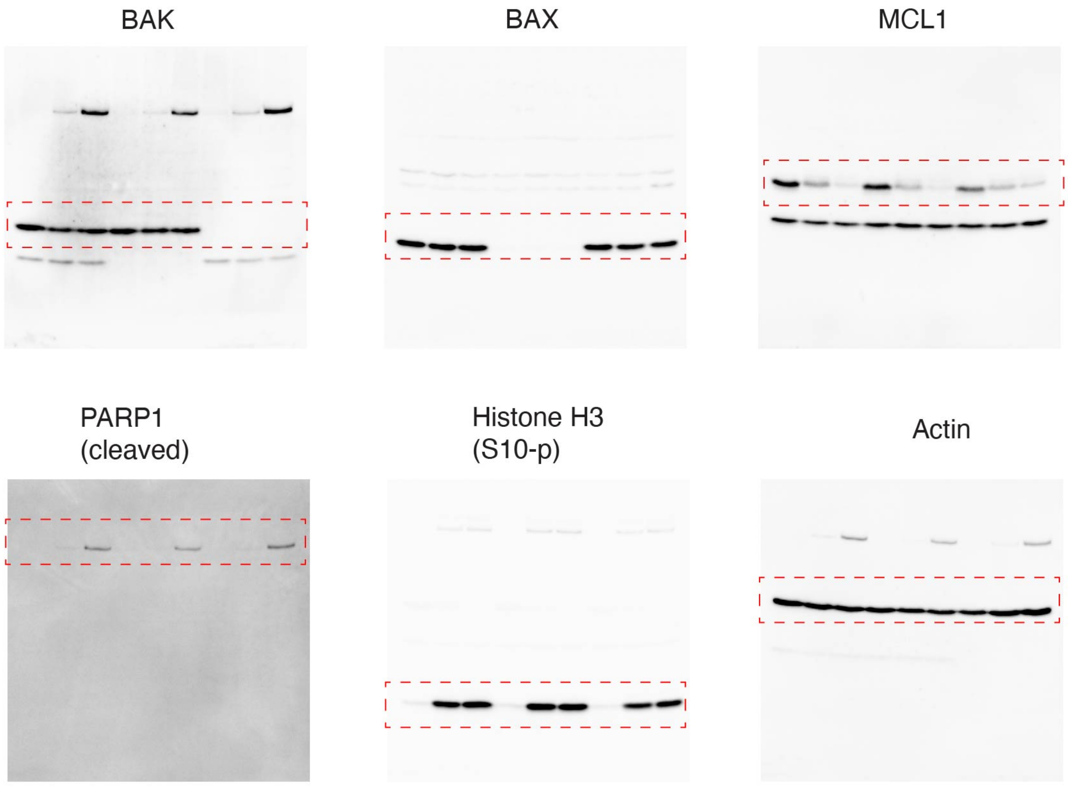

Fig S9C

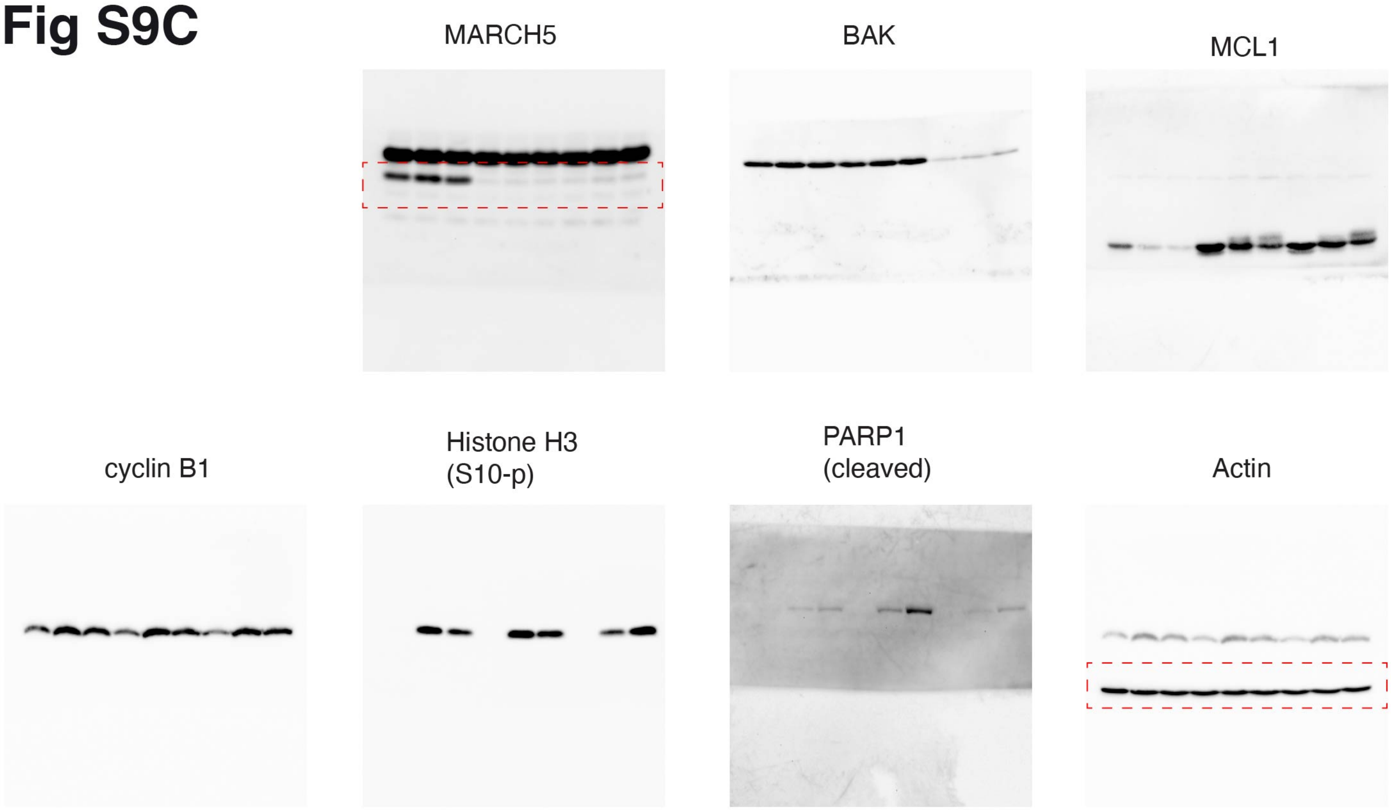

Original Western blots of Figure S9

Fig S8

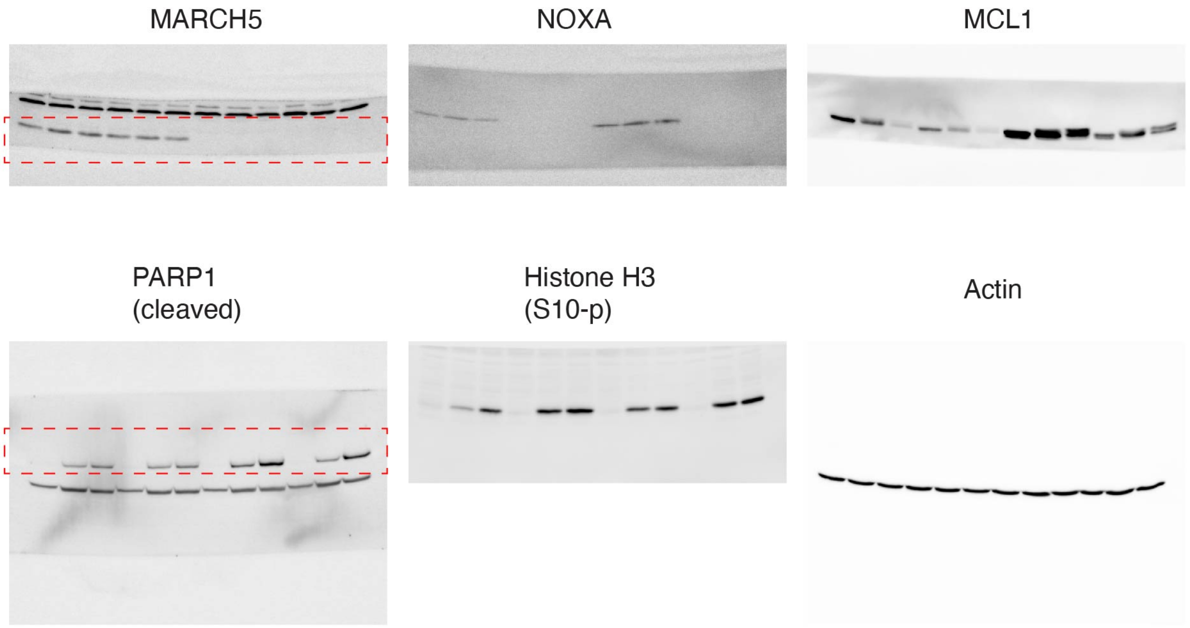

Original Western blots of Figure S8

Fig S10

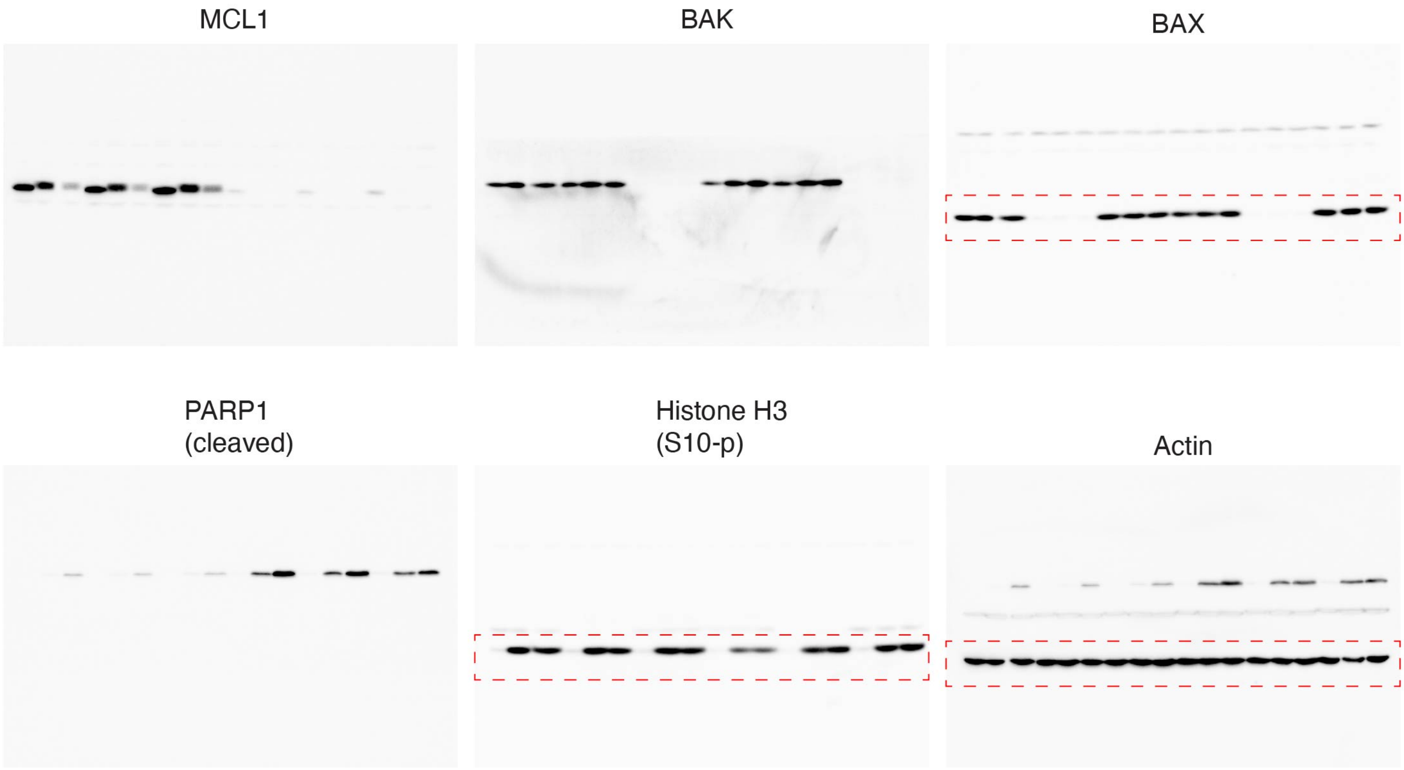

Original Western blots of Figure S10
